# A review on bio-electro-Fenton systems as environmentally friendly methods for degradation of environmental organic pollutants in wastewater

**DOI:** 10.1039/d1ra08825d

**Published:** 2022-02-10

**Authors:** Fatemeh Soltani, Nahid Navidjouy, Mostafa Rahimnejad

**Affiliations:** Student Research Committee, Urmia University of Medical Sciences Urmia Iran; Department of Environmental Health Engineering, Urmia University of Medical Sciences Urmia Iran n.navidjouy@gmail.com +98 9143489617; Biofuel and Renewable Energy Research Center, Department of Chemical Engineering, Babol Noshirvani University of Technology Babol Iran

## Abstract

Bio-electro-Fenton (BEF) systems have been potentially studied as a promising technology to achieve environmental organic pollutants degradation and bioelectricity generation. The BEF systems are interesting and constantly expanding fields of science and technology. These emerging technologies, coupled with anodic microbial metabolisms and electrochemical Fenton's reactions, are considered suitable alternatives. Recently, great attention has been paid to BEFs due to special features such as hydrogen peroxide generation, energy saving, high efficiency and energy production, that these features make BEFs outstanding compared with the existing technologies. Despite the advantages of this technology, there are still problems to consider including low production of current density, chemical requirement for pH adjustment, iron sludge formation due to the addition of iron catalysts and costly materials used. This review has described the general features of BEF system, and introduced some operational parameters affecting the performance of BEF system. In addition, the results of published researches about the degradation of persistent organic pollutants and real wastewaters treatment in BEF system are presented. Some challenges and possible future prospects such as suitable methods for improving current generation, selection of electrode materials, and methods for reducing iron residues and application over a wide pH range are also given. Thus, the present review mainly revealed that BEF system is an environmental friendly technology for integrated wastewater treatment and clean energy production.

## Abstract 2

Bio-electro-Fenton system is a promising technology for the environmental organic pollutants degradation and bioelectricity generation.

## Graphical abstract

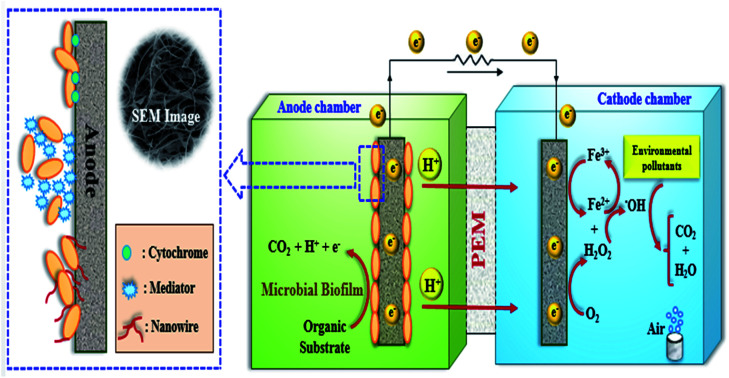

## 1. Introduction

The population growth and the rapid development of industry and agriculture despite their numerous benefits have caused problems for human societies and the environment. Increasing emissions of pollutants that result from fossil fuel consumption, global climate changes, energy shortages, and environmental pollution are grave global problems with negative impacts on the environment.^1–3^ Also, the demand for energy is progressively increasing around the world.^4^ Therefore, controlling environmental pollution and dealing with the energy crisis are matters of concern in many countries. Moreover, water pollution is a major concern due to the problems which arise from the removal of environmental organic pollutants from wastewater.^5–7^ Effluents, which stem from agricultural, domestic and industrial activities, are the main sources of natural water pollution due to the release of toxic and resistant organic pollutants. Therefore, wastewater treatment is necessary to prevent water pollution, protect water resources, and preclude the spread of diseases.^8,9^ A number of common physical, chemical, and biological methods are used to treat wastewater containing persistent environmental pollutants. These methods include coagulation/flocculation, membrane separation technology, ion exchange, aerobic and anaerobic biological treatment and adsorption.^10–14^ Nonetheless, despite the advantages of these methods, their high need for energy and chemicals, high operational costs, insufficient removal of toxic environmental organic pollutants, and time-consuming biological processes are their general limitations regarding the removal of toxic and resistant organic pollutants.^15–18^

Advanced oxidation processes (AOPs) have emerged as effective technologies to degradation of organic pollutants.^17,19,20^ Photocatalysis,^21,22^ ozonation,^23,24^ photo-Fenton,^25,26^ photo-electro-Fenton,^27,28^ electro-Fenton (EF),^29,30^*etc.* are high-performance advanced oxidation processes. Among these technologies, the electro-Fenton process is commonly used as an electrochemical advanced oxidation process (EAOP), based on the generation of potent hydroxyl radical (˙OH) to oxidize resistant organic matter in wastewater.^31,32^ During this process, oxygen is transferred to the solution phase and the reduction of two oxygen electrons occurs continuously at the cathode and leads to the electrochemical production of hydrogen peroxide (H_2_O_2_) in acidic conditions. Then, the produced H_2_O_2_ reacts with the ferrous iron to produce homogeneous ˙OH and the chemical oxidation of the organic pollutants resistant to CO_2_, water, and mineral salts begins.^13,31,33,34^ Despite its advantages such as without of the need for storage and transportation of H_2_O_2_, reduced sludge production compared with the Fenton process, and high efficiency regarding the removal of a wide range of environmental organic contaminants due to the need for high electricity, this process requires high operational costs, which limit its practical application.^35–37^ Furthermore, in other technologies such as photocatalysis processes under UV-light irradiation,^22,38^ an external energy source has been used to decompose organic pollutants, which this has attracted the researchers' attention to the use of photocatalytic processes under sunlight irradiation in recent years.^39–41^ In these processes with potential applications, different photocatalysts with high photocatalytic activities are used.^40,42^

In recent years, research in the field of developing novel methods with low energy consumption and cost, which can produce energy and remove the environmental organic pollutants, has been of great importance and this issue has received much attention of the scientists.^43–45^ In this regard, bio-electrochemical systems (BESs), such as microbial fuel cell (MFC) and microbial electrolysis cell (MEC) are considered to be promising approaches for the degradation of biorefractory contaminants and simultaneous electricity generation.^16^

As an innovative and environmentally friendly method, a MFC system can convert energy, which is stored in chemical bonds of organic compounds, directly into electrical energy through a catalytic reactions by electrogenic microorganisms.^46–51^ In MFCs, the released electrons from the anodic oxidation of biodegradable organic matter (simple and complex substrates, complex organic waste, and organic matter in wastewater) as electron donors are used (eqn (1)) to bioelectricity production.^52–55^

1 C_*x*_H_*y*_O_*z*_ + (2*x* − *z*)H_2_O → *x*CO_2_ + (*y* + 4*x* − 2*z*)H^+^ + (*y* + 4*x* − 2*z*)e^−^

Especially, direct recovery of electrical energy is possible in MFC technology. Moreover, high effluent quality and low environmental footprint can be attained due to the combination of electrochemical and biological processes.^56^

**Fig. 1:**
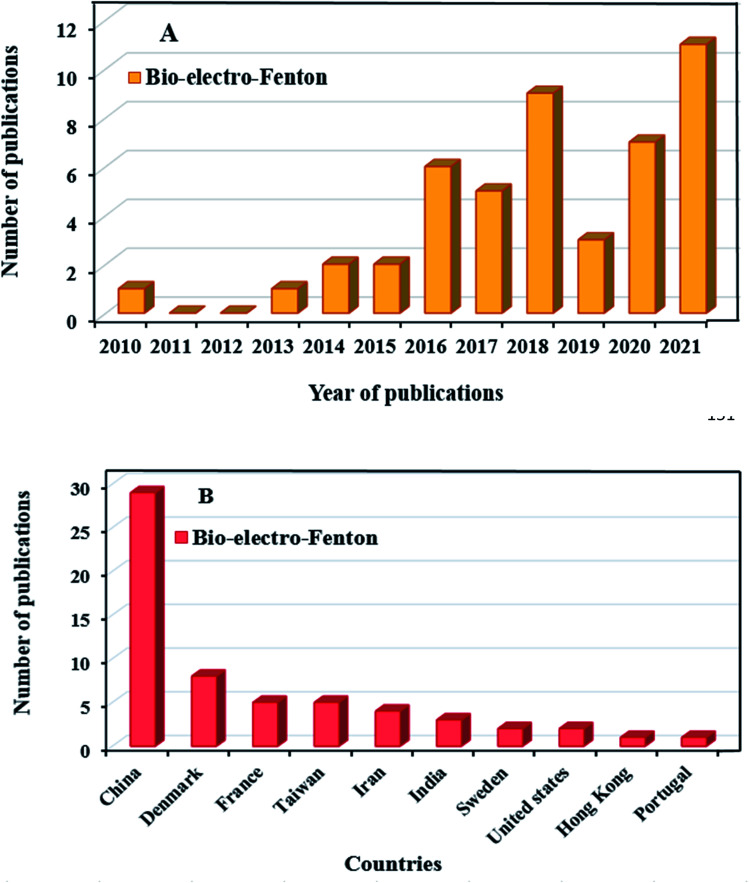
Number of publications of the bio-electro-Fenton systems by year of publications (A) and country (B) (Scopus data, accessed on October 15, 2021).^60^

**Fig. 2:**
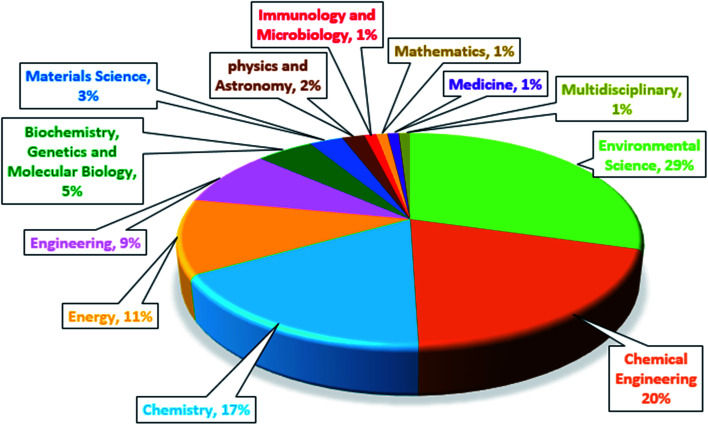
Publications of the bio-electro-Fenton systems by subject area.^60^

Recently, research on the MFCs combined with advanced oxidation processes has been carried out. The bio-electro-Fenton (BEF) system as an environmentally friendly method has been used to effective treatment of the effluents, which contain persistent organic compounds.^16,57^ The BEF is a combined process of MFC system with EF that it was first proposed by Ni and Zhu in 2009 to generate the energy and *p*-nitrophenol degradation simultaneously.^35,58^ The BEF system has several common features with EF process in terms of cathodic reaction and reactor configuration. The major factor that makes BEF process more cost-effective than the traditional EF process, is that the electrical energy in the BEF system is generated from organic matter oxidation, instead of using power input.^59^ As shown in Fig. 1A, BEFs are still in the early stages of development, and growing research is being done in this concept. More specifically, Fig. 1B shows the number of publications regarding BEF systems by country. China has grown significantly in this area and has the largest number of publications among other countries, applying this novel technology for different wastewater treatment along with power generation. A general research of subject areas shows that the highest research interests are in the subject areas of Environmental Science, Chemical Engineering and Chemistry, respectively, and the number of publications in these subject areas has increased (Fig. 2).^60^

**Fig. 3:**
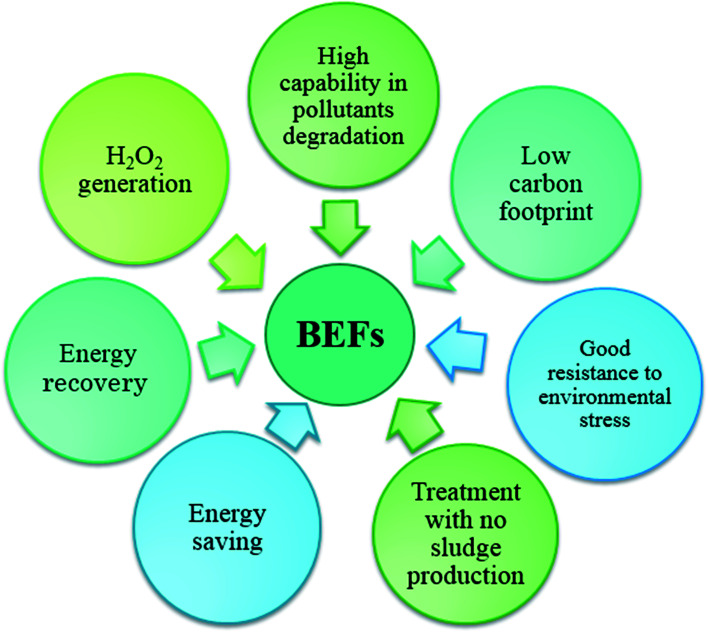
Potential benefits of BEF systems.

In BEF systems, during the biological decomposition of organic carbon in the anode chamber by electro active microorganisms that produce electricity, the oxidation of environmental organic pollutants in the cathode chamber is done by Fenton-based reactions.^33,61,62^ The BEF systems could have significant benefits because of its unique features (Fig. 3).^53^ In recent years, the feasibility of the BEF system to treat of a wide range of synthetic wastewaters, which contains a variety of environmental organic compounds, such as dyes,^63–67^ industrial pollutants,^68–70^ and pharmaceutical compounds^71–73^ has been demonstrated. In this review, the mechanisms and configuration of the BEF systems are described. Some important operating parameters affecting the BEF systems performance are also introduced. Furthermore, an overview of the application of BEF system to the removal of persistent organic pollutants in the environment and energy generation is specifically offered to highlight the use of this system as a new sustainable method. Finally, some challenges and possible future prospects are presented that will be useful for the development of BEFs in the future. The aims of the present review are to introduce the BEF system as an efficient technology for wastewater treatment and clean energy production, as well as to provide a reference for researchers in developing more efficient BEF systems.

## 2. Mechanism, configuration and features of the bio-electro-Fenton system

**Fig. 4:**
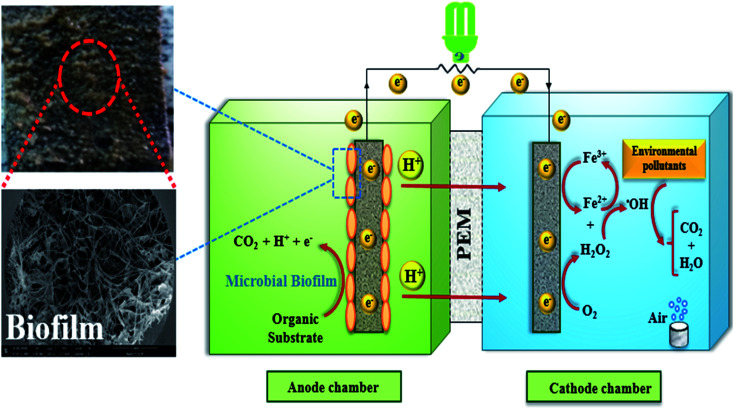
Schematic diagram of bio-electro-Fenton system with Microbial biofilm layer.

The BEF system is an efficient and energy-saving bio-electrochemical technology that is highly effective in wastewater treatment that contains toxic and non-biodegradable contaminants. It can directly convert the chemical energy, which is stored in biodegradable organic matters, into electrical energy using microbial catabolism.^74,75^ The schematic diagram of a BEF system for organic pollutants degradation in wastewater is shown in Fig. 4.

In general, the main components of a BEF system include the anode chamber, the cathode chamber, the membrane, and the substrate. The anode chamber consists of the anode electrode, the substrate, and the microbial culture. Moreover, the cathode chamber consists of the cathode electrode and the electron acceptor (*e.g.*, oxygen with high oxidation potential), and environmental persistent organic pollutants.^47,62^ The anode chamber must be anaerobic because the presence of oxygen disrupts the anaerobic bacteria activities and limits the bioelectricity generation. Nonetheless, the cathode chamber is aerated continuously to provide the dissolved oxygen needed to perform the reactions.^1,76^ In general, the anode and cathode chambers are separated from each other by a membrane.^62,77^ Nafion membrane is regarded to be the most widely used proton exchange membrane in the structure of two-chamber MFCs.^78^

**Fig. 5:**
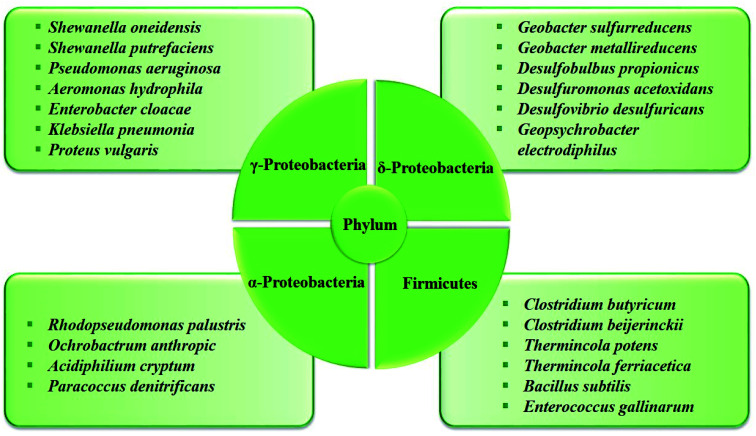
The electrogenic microorganisms in BESs, summarized from the corresponding ref. 3 and 82.

In the anaerobic anode chamber, the accumulation of electrogenic bacteria on the anode electrode causes the formation of bio-anodes and these bacteria act as biocatalysts and electricity generators.^43,79,80^ Among electrogenic bacteria, *Geobacter* and *Shewanella* are well-known species with capable of extracellular electron transfer (EET) that attach to the electrode and form a biofilm layer.^81^ A list of some typical electrogenic microorganisms is shown in Fig. 5.

**Fig. 6:**
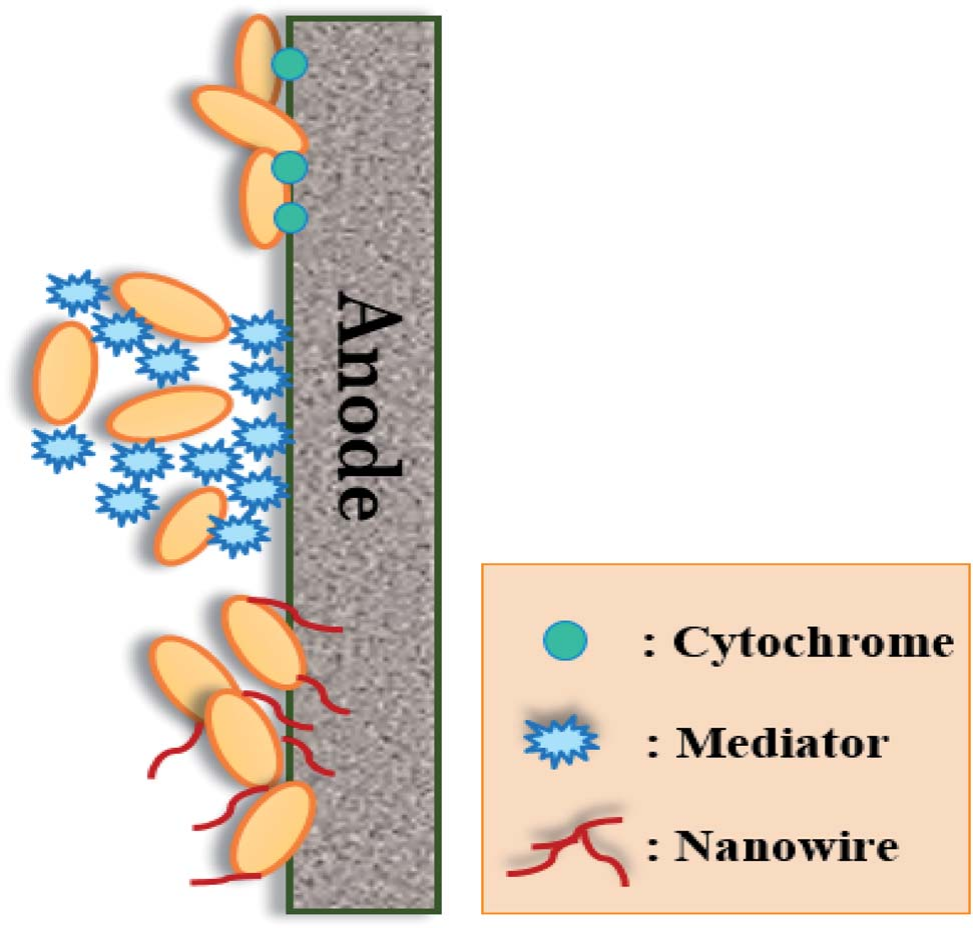
The extracellular electron transfer mechanisms of microbial bioanode.

The electrogenic bacteria can transport electrons to electrode surfaces by different electron-transferring mechanisms, including direct and mediated electron transfers (Fig. 6). Direct electron transfer are usually occurred *via* outer membrane redox-active proteins such as c-type cytochromes or conductive pili as “nano-wires”, whereas mediated electron transfer uses endogenous or exogenous electron mediators (*e.g.*, flavin).^3,48^

**Fig. 7:**
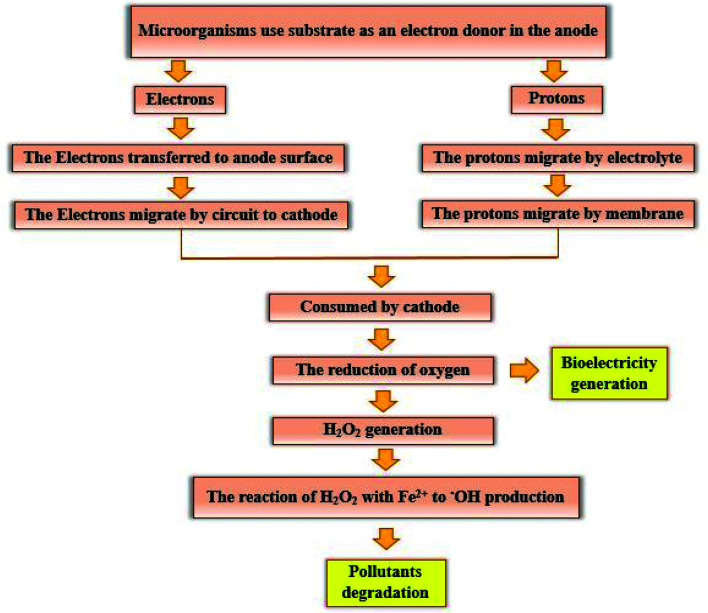
Schematic representation of the pollutants degradation mechanism and bioelectricity generation.

The electrogenic bacteria oxidize the substrate (either complex wastewaters or simple substrates *e.g.* glucose) as an electron donor to produce electrons and H^+^ ions (eqn (2)), thereby the electrons transfer to the electrode surface and H^+^ ions is also diffuse through the membrane into the cathode. At the same time, electrons are also transferred from the anode to the cathode through an external resistor, to be used to generate electricity and to reduce the dissolved oxygen.^3,83,84^ H_2_O_2_ is continuously produced in site (eqn (3)) due to the reduction of two oxygen electrons in the cathode chamber.^85–87^ Moreover, Cathodic Fenton-based reactions take place. H_2_O_2_ reacts with Fe^2+^ ions under acidic conditions and produces ˙OH (eqn (4) and (5)).^88–90^ ˙OH with high oxidation potential can result in the non-selective degradation of environmental organic pollutants (eqn (6)) (Fig. 7).^65,91^ Meanwhile, in this process, Fe^2+^ is continuously produced by reducing Fe^3+^ (eqn (7)).^57,92^

Anodic reaction:

2 C_6_H_12_O_6_ + 6H_2_O → 6CO_2_ + 24H^+^ + 24e^−^

Cathodic reactions:

3 O_2_ + 2H^+^ +2e^−^ → H_2_O_2_

4 Fe^2+^ + H_2_O_2_ → Fe^3+^ + ˙OH + OH^−^

5 Fe^3+^ + H_2_O_2_ → Fe^2+^ + HO_2_˙ + H^+^

6 Environmental organic pollutants + ˙OH → oxidation products

7 Fe^3+^ + e^−^ → Fe^2 +^

It has been proven that the BEF process can simultaneously produce bioenergy and decompose the environmental persistent organic pollutants in effluent without the need for external energy.^58,69,75,78^ This process has attracted many researchers' attention as a cost-effective and efficient treatment process that produces *in situ* H_2_O_2_ electrochemically instead of its commercial application in the cathode chamber.^93,94^ H_2_O_2_ concentration is considered to be the most important parameter in Fenton oxidation and is affected by the types and properties of cathode materials.^94^ The amount of H_2_O_2_ generation in different bio-electrochemical systems is shown in Table 1. In 2010, *in situ* production of H_2_O_2_ was proved successful in a MFC-Fenton system using carbon-based materials.^35^ Chen *et al.*^95^ demonstrated that H_2_O_2_ concentration reached 196.50 mg L^−1^ in MFC equipped with a three-dimensional electrode made of activated carbon particles. Li *et al.*^96^ made a carbon black and graphite hybrid air cathode MFC for H_2_O_2_ generation. The maximum H_2_O_2_ yield was obtained 11.9 mg L^−1^ h^−1^ cm^−2^ when the optimal mass ratio of carbon black to graphite was 1 : 5. Xu *et al.* observed that the H_2_O_2_ concentration in the BEF system with cathodic electrodes of Fe@Fe_2_O_3_/NCF (non-catalyzed carbon felt) and NCF reached 1.21 and 0.09 mg L^−1^ within 10 h, respectively.^33^

**Table 1:** The H_2_O_2_ production in bio-electrochemical systems

Reactor	Anode material	Cathode material	Current density	H_2_O_2_ concentration	Ref.
MFC	Carbon felt	Carbon felt	0.76 mA	0.11 mmol L^−1^	58
MFC	Carbon felt	Graphite particle electrode (GPE)	18.41 A m^−3^	196.50 mg L^−1^	95
BEF	Carbon fiber brush	Graphite plate and carbon paper	0.49 A m^−2^	180 mg L^−1^	98
BEF	Graphite plate	Fe@Fe_2_O_3_/graphite	550.21 mA m^−2^	0.62 mg L^−1^	78
BEF	Graphite felt	Fe@Fe_2_O_3_/graphite felt	252.22 mA m^−2^	135.96 μmol L^−1^	68
BEF	Carbon felt	Carbon felt	1.7 A m^−2^	1400 mg L^−1^	99
BEF	Carbon felt	FeVO_4_/carbon felt	2.6 A m^−3^	0.05 mmol L^−1^	100
MFC	Carbon brush	Graphene oxide	5 A m^−2^	273 mg L^−1^	101
BEF	Graphite felt	Fe–Mn/graphite felt	1156.25 mA m^−2^	128.65 μmol L^−1^	102

The amount of resistance is another factor that can affect the H_2_O_2_ production in BEF system. The study results of Fu *et al.* showed that low external resistance was favorable for H_2_O_2_ production in the MFC and increasing external resistance had a negative effect on H_2_O_2_ production. Thus, H_2_O_2_ concentration was 78.85 mg L^−1^ after 12 h with an external resistance of 20 Ω.^97^

Other operational parameters such as the substrate type, operating mode (continuous and batch), and cathodic current density were shown to influence H_2_O_2_ generation in BEF. Specifically, Wang *et al.* reported that the maximum H_2_O_2_ concentration in glucose-fed MFC system and the acetate-fed MFC system were 0.36 and 0.08 mg L^−1^, respectively. This may be due to the high community diversity in glucose-fed MFC. In addition, it was demonstrated that in both continuous and batch modes, the H_2_O_2_ generation had a rising trend at first but subsequently it declined.^93^ A more production of H_2_O_2_ can be possible at a relatively higher current density.^73^ Zhuang *et al.*^63^ produced the H_2_O_2_ in a BEF system with a concentration of around of 0.02 and 0.01 mM under short- and close-circuit conditions, respectively, because the current density in short circuit conditions was 2.1–2.6 times higher than the current density in closed circuit conditions.

The possible application of this technology to achieve potential wastewater treatment and large-scale electricity generation is promising in the near future. Therefore, it is necessary to use new and developed BEF processes for practical applications.^62,103^ In the following sections, different types of cathodic materials, anodic materials, membranes, and sources of iron catalysts will be introduced as important operational parameters affecting the performance of BEF system.

### 2.1. Cathodic materials

Using a suitable electrode in the structure of the BEF system can improve the system's performance. Since the production of H_2_O_2_ through oxygen reduction plays an important role in the decomposition of resistant organic pollutants at the cathode, the type and structural properties of cathode electrode materials affect the H_2_O_2_ production. Cathodic materials must have the following specifications: (a) catalytic properties for O_2_ reduction, (b) high surface area and porosity, (c) high stability and durability against corrosion, (d) good electrical conductivity, (e) having sites of redox reaction, (f) low cost and convenient access.^53,104,105^

To improve and enhance the oxygen reduction reaction to produce H_2_O_2_, various carbon-based materials have been extensively tested as cathodic electrodes, including carbon felt (CF),^106^ graphite,^98^ carbon nanotubes (CNT),^35^ gas-diffusion electrodes,^107^*etc.* According to studies, CF is one of the most common and widely used cathode materials in BEFs. CF has a high specific surface area, high stability, and flexibility and has good electro-catalytic properties for the reduction of oxygen into H_2_O_2_.^105,108^ In addition, its cost is relatively low, and the mechanical strength, depending on the thickness of the material, is high.^52^

Fe-based carbonaceous materials have also been widely studied as composite cathodic electrodes due to their high performance in producing large quantities of H_2_O_2_. Three dimensional carbonaceous materials, such as active carbon felt (ACF), CF and graphite felt are the common materials for preparing Fe-based carbonaceous electrodes.^104^ For example, Fe-based carbonaceous cathode electrodes such as CF/γ-FeOOH,^69^ Fe@Fe_2_O_3_/graphite felt,^68^ Fe@Fe_2_O_3_/NCF,^33^ CNT/γ-FeOOH,^35^ and Fe_2_O_3_/ACF,^109^ have been successfully used to decompose resistant organic pollutants.

### 2.2. Anodic materials

The fundamental and structural properties of anodic materials directly affect the performance of the BEF system *via* their effect on the adhesion of microorganisms and the through effectiveness of electron movement from microorganisms to electrodes. Therefore, selecting the appropriate anode materials and modifying them is essential to increase the system's power output.^110^ To achieve higher performance of BEFs, the material of anodic electrode must have good biocompatibility, a large surface area, good resistance against corrosion, chemical stability and acceptable and appropriate cost. Among the properties of anodic materials, surface area, pore structure, and surface hydrophilicity have important effects on anodic biofilm formation.^3^

The most widely used materials in the anode are made of carbon material. Carbon fiber, CF, graphite, and granular graphite are the most common anode materials of BEFs.^105^ CF is a porous, inexpensive, and highly conductive three-dimensional carbon material whose porous structure creates a wide area for the growth of exoelectrogenic microorganisms and thus the proper transmission of electrons.^111^ With carbonated anode materials, the maximum power density of 2437 and 2110 mW m^−2^ (90% COD removal) were obtained using anodic electrodes of CF and carbon brush, respectively.^112,113^ Also, the simplest materials as anodic electrodes are graphite plates or rods, which are cheap, easy to carry and have a certain surface area.^105^ For example, the maximum power density of 1771 mW m^−2^ was produced using graphite plates in the MFC system.^114^

**Fig. 8:**
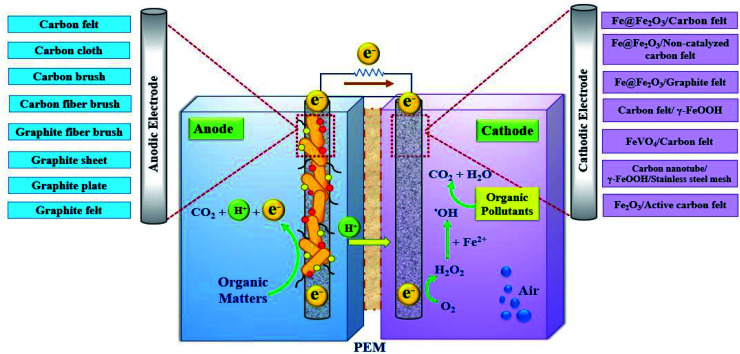
The anode and cathode materials employed in BEF systems.

In recent years, researchers have sought to increase the bioelectrocatalytic ability of carbon materials through various types of modification and fabrication techniques to increase bacterial cell adhesion and electron transfer.^48^ These surface changes include the use of carbon nanoparticles, metal nanoparticles, and polymer deposition.^115–117^ In a study by Park *et al.*, an anode made with a combination of CNT and iron (II, III) oxide (Fe_3_O_4_) in a mediator-less MFC showed a power density of 830 mW m^−2^. The attachment of Fe_3_O_4_ to CNTs creates a multilayered network that increases microbial growth and electron transfer.^118^Fig. 8 shows the different types of anode and cathode electrodes used in BEFs.^53^

### 2.3. Membranes

Membranes are used to separate anode solution from the cathode and transfer protons from anode to cathode. Membranes prevent the passage of substrate and microorganisms from the anode to the cathode and also prevent the passage of oxygen from the cathode to the anode. However, their main problem is their scarcity and high price.^76,119^ Membranes are an important part of the structure of BEFs that greatly affect system performance. The most commonly used membranes are ion-exchange membranes which include proton exchange membrane (PEM), cation exchange membrane (CEM), and anion exchange membrane (AEM).^53^ PEMs allow protons to enter the cathode chamber. Nafion-117 is the most widely used membrane due to the highly selective permeability of protons in BEFs and is a type of PEM. However, the side effects of transferring other cations during MFC startup are inevitable even with Nafion; and it preferably conducts other positive ions that are approximately 10^5^ times higher than concentrations of protons in the solution. Despite researchers' efforts to find cheaper and more durable alternatives, Nafion remains the best choice. Concerning energy production, PEMs are also superior to CEMs.^1,82^ Min *et al.*^120^ compared the performance of a PEM membrane and a salt bridge in an MFC. The output power was 2.2 mW m^−2^ using the salt bridge; which was once less than the power obtained using Nafion. CEMs are less expensive and structurally more stable than PEMs (such as Nafion-117). Also, CEMs show higher internal resistance than Nafion due to the transmission of all cations through the membrane.^108^ This type of membrane causes difficulty in maintaining low pH in the cathode chamber that can disrupt the Fenton reaction.^53^

Due to the limitations of CEM, researchers proposed AEM, which uses carbonate and phosphate as pH buffer to improve proton transfer. AEM consists of positively charged ions (*e.g.*, –PR_3_^+^, –SR_2_^+^, COO^−^, –NH_3_^+^) that attach to the membrane and transfer anions through it. Kim *et al.* obtained a higher power density of about 0.61 W m^−2^ using AEM compared to CEM, which had a power density of 0.48 W m^−2^.^121^ However, AEMs have not been widely used in BEF processes as much as CEM. AEMs are more prone to deformation, which may significantly increase the internal resistance of the system; hence they cannot be a good option for long-term operation.^108^ Advantages and disadvantages of Ion exchange membranes are given in Table 2.^76,122–125^ The bipolar membrane (BPM), composed of two monopolar membranes (CEM and AEM) is alternative separator used in MFCs where protons and hydroxide ions are conducted. BPM can be used to help maintain the low pH of the catholyte without the need to add additional doses of acid. Metal materials such as graphite and stainless steel have been widely used for bipolar plate membranes.^53,76^

**Table 2:** Advantages and disadvantages of Ion exchange membranes

Membrane	Advantages	Disadvantages
CEM/PEM	- Strong and stable in oxidative and reductive environment	- Transport of cations more than protons (PEM)
	- High chemical and mechanical stability	- High cost (PEM)
	- High proton transfer ability (Nafion)	- pH imbalance (CEM)
	- Prevent the transfer of oxygen, substances and minerals from the anode to the cathode chamber	- Membrane chemical and biological fouling (Nafion)
AEM	- Usage of cheaper materials	- More sensitive to deformation
	- Use carbonate and phosphate as pH buffer to facilitate proton transfer	

### 2.4. Homogeneous and heterogeneous iron catalysts

**Fig. 9:**
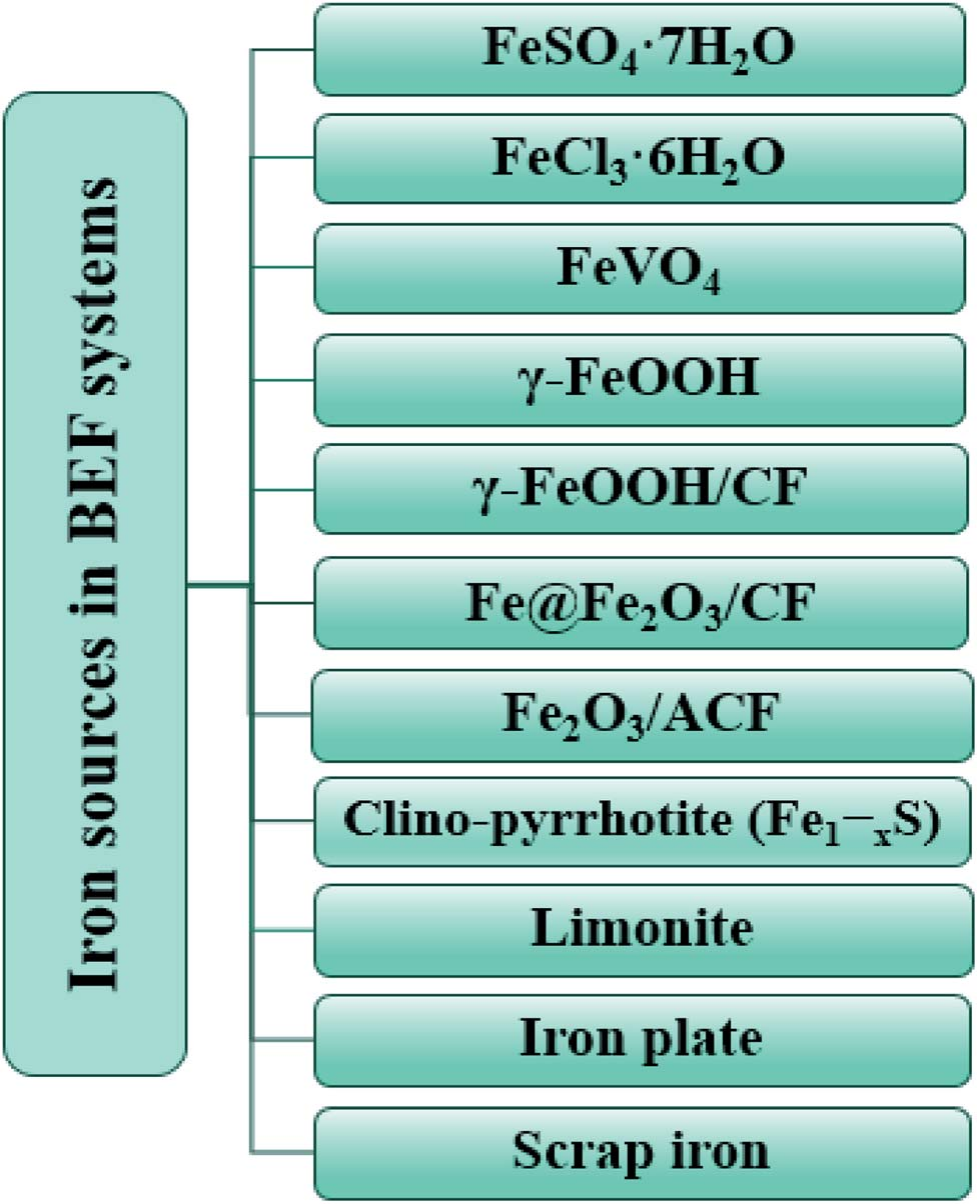
The iron sources used in BEF systems.

Iron catalyst is a main factor affecting the performance of BEF system. ˙OH is produced by the Fenton's reaction between Fe(ii) and H_2_O_2_ (eqn (4)). Later, ˙OH reacts with resistant organic pollutants, leading to their decomposition (eqn (6)).^81^ There are different types of homogeneous or heterogeneous iron sources that are used as Fenton catalysts in BEFs. Fig. 9 presents the iron sources used in BEF systems. Sources of homogeneous iron include iron(iii) chloride hexahydrate (FeCl_3_·6H_2_O) and iron(ii) sulfate heptahydrate (FeSO_4_·7H_2_O), which are relatively cheaper than heterogeneous types of iron sources.^99,126^

In Fenton homogeneous processes, when large quantities of iron are used, the residual iron produces some sludge, so that the removal of sludge at the end of wastewater treatment is operationally costly and it needs a lot of chemicals.^127,128^ In addition, homogeneous Fenton reactions are carried out under acidic conditions, which require additional pH adjustment steps. Recently, researchers have used heterogeneous iron salts as Fenton catalysts.^129–131^ The performance of these oxides depends on their physicochemical properties.^132^ Magnetite iron mineral with chemical formula Fe_3_O_4_, cubic crystal structure and iron content of 73%, is the most common iron-oxide based Fenton catalyst with high catalytic activity that has a high ˙OH release power through Fenton's reaction. Furthermore, it contains divalent and trivalent iron ions. Other physicochemical properties of this iron mineral include the presence of octagonal sites on the crystal surface, high magnetic properties, and high dissolution rate.^108,132,133^ Another iron mineral is goethite (α-FeOOH), which with an orthorhombic crystal system, a surface area of 8–200 m^2^ g^−1^ and iron content of 63%, contains ferric iron and is relatively inexpensive and environmentally friendly. It is also used in a wide range of pH.^108,134,135^ Hematite (α-Fe_2_O_3_), with a trigonal crystal system, is also an iron oxide which has a density of 5.26 g cm^−3^, a surface area of 100–400 m^2^ g^−1^, and an iron content of 61–70%.^108,132,136,137^ Ferrites are ceramic-like materials with magnetic properties. Ferrites are composed of iron oxide and one or more other metals in chemical combination. These materials have excellent adsorptive and catalytic properties. M-type hexagonal ferrites MFe_12_O_19_ (M = Ba, Pb, Sr) have been noted for their high magnetic properties, great chemical stability, corrosion resistance, good permeability and low cost, so their application is increasing.^73,138,139^ Complete degradation of tetracycline, sulfamethoxazole and tylosin (74.8–87.2% TOC) was performed using M-type strontium hexaferrite magnetic nanoparticles as a heterogeneous iron catalyst.^73^

In recent years, many studies have used these iron minerals as composite electrodes in heterogeneous Fenton processes of BEFs which can be referred to designed composite electrodes such as Fe@Fe_2_O_3_/CF,^61^ γ-FeOOH/CF,^69^ CNT/γ-FeOOH/CF,^35^ PPy (Polypyrrole)/AQDS (anthraquinone-2,6-disulfonate)/CF^140^ and Fe_2_O_3_/ACF.^109^ In a study by Xu *et al.*, maximum degradation of estrogens was obtained using the Fe@Fe_2_O_3_/NCF composite electrode in the BEF, and maximum power density of 4.35 W m^−3^ was produced.^33^ According to reports, if these heterogeneous iron sources are used, the production of iron sludge will be greatly reduced and the operating pH amplitude expands.^129^

## 3. Application of bio-electro-Fenton system for the environmental organic pollutants degradation

**Fig. 10:**
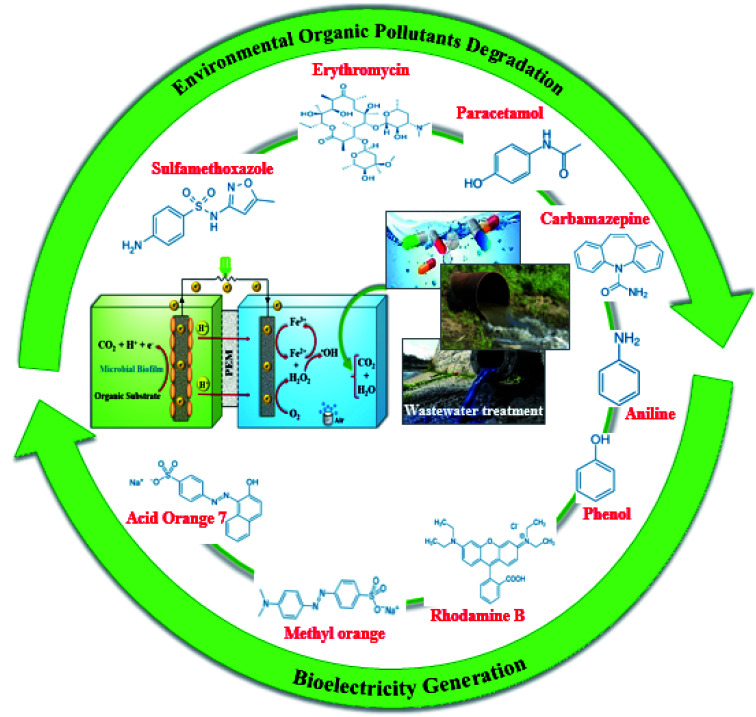
Performance of environmental organic pollutants degradation in BEF systems.

BEF technologies are a promising approach to environmental protection and water reuse. These technologies have different environmental applications for treating a wide range of real wastewater and environmental organic pollutants such as different types of industrial dyes, pharmaceuticals, and emerging pollutants from different sources and industries.^33,35,57,62,65,68,73,90^Fig. 10 summarizes the organic compounds that have been decomposed by the BEF technologies.

### 3.1. PPCPs/ECs

In recent years, the widespread presence of emerging contaminants (ECs) in water and wastewater resources has caused great concern due to their negative effects on the ecosystem.^141^ Most of them do not have any regulatory standards and can potentially threaten the aquatic life and environment.^142^ These effluents contain various ECs, including pharmaceuticals and personal care products (PPCPs), disinfectants, detergents, perfumes, insecticides, steroids, hormones, industrial additives, and many chemicals. Many of these compounds are known as endocrine disrupting compounds (EDCs).^141,143^ Therefore, due to the toxic effects of EDCs on the environment, human health, and drinking water supply, the removal of these compounds from water supplies and wastewater using advanced treatment methods is essential.^144,145^ Recent studies on innovative BEF systems have been developed for treating wastewaters, which contains ECs and pharmaceutical compounds including *P*-nitrophenol, phenol, bisphenol A, sulfamethazine, triclocarban, carbamazepine, non-steroidal anti-inflammatory drugs, erythromycin, paracetamol, *etc.* (Table 3).^57,58,75,93,146,147^ In this regard, the application of BEF systems with similar or different structure in terms of the type of electrode, membrane, iron catalyst, substrate and reactor structure has been investigated in various studies.^90,108,146^

**Table 3:** Literature review on application of BEF systems for PPCPs/ECs degradation

Target PPCPs/ECs	Membrane	Anodic electrode	Cathodic electrode	Removal efficiency		Operation time	Operation conditions	Max. power generation	Ref.
				Anode	Cathode				
*P*-Nitrophenol (PNP)	PEM	Carbon felt	Carbon felt	—	100% (complete degradation)	12 h	External resistance (ER): 1000 Ω, anodic inoculum: anaerobic sludge, substrate type: glucose, the volume of each chamber: 300 mL, cathodic pH: 3, catholyte: (10 mM PNP and 0.2 M Na_2_SO_4_), iron catalyst: 10 g scrap iron	143 mW m^−2^	58
*P*-Nitrophenol	PEM (Nafion-117)	Carbon felt	Carbon felt	—	96%	6 h	ER: 20 Ω, anodic inoculum: anaerobic sludge, substrate type: sodium acetate, the working volume of two chambers: 170 mL, cathodic pH: 2, catholyte: (0.25 mM PNP and 1 M Na_2_SO_4_), iron catalyst: 112 mg limonite, air flow rate: 100 mL min^−1^, temperature: 35 °C	237.5 mA m^−2^	106
Phenol	Membrane less	Iron plate	Carbon felt	—	100% (complete degradation)	22 h	ER: 1000 Ω, substrate type: sodium acetate, total volume of cell: 28 mL, total volume of EF: 80 mL, cathodic pH: 3, catholyte: (1 mM phenol and 50 mM KH_2_PO_4_), temperature: 25–30 °C	1746 ± 100 mW m^−2^	147
17β-Estradiol (E2), 17α-ethynyl-estradiol (EE2)	PEM	Carbon felt along with graphite granules	Fe@Fe_2_O_3_/NCF	—	81% E2, 56% EE2	10 h	ER: 1000 Ω, anodic inoculum: anaerobic sludge, substrate type: glucose, the liquid volume of each chamber 75 mL, cathodic pH: 3, catholyte: (20 μg L^−1^ of E2 and EE2 and 0.1 M NaCl), air flow rate: 100 mL min^−1^, temperature: 30 °C	4.35 W m^−3^	33
Arsenite(iii)	CEM	Carbon felt	Carbon felt/γ-FeOOH	—	98.5%	72 h	ER: 1000 Ω, anodic inoculum: Shewanella putrefaciens SP200, substrate: lactate, the volume of each chamber: 75 mL, catholyte: (1 mg L^−1^ AS(iii), 100 mM PBS (pH: 7)), air flow rate: 100 mL min^−1^, temperature: 30 °C	135.3 mW m^−2^	69
Paracetamol (PAM)	PEM (Nafion-117)	Graphite felt	Graphite plate	—	70%	9 h	ER: 180 Ω, anodic inoculum: anaerobic sludge, substrate type: sodium acetate, the working volume of two chambers: 324 mL, cathodic pH: 2, catholyte: (10 mg L^−1^ PAM, 5850 mg L^−1^ NaCL and 5 mg L^−1^ FeSO_4_), air flow rate: 1.0 L h^−1^, temperature: 25 °C	217.27 ± 23.24 mW m^−2^	75
Triphenyltin chloride (TPTC)	PEM (Nafion-117)	Graphite felt	Fe@Fe_2_O_3_/graphite felt	—	78.32 ± 2.07%	101 h	ER: 2 KΩ, anodic inoculum: *S. oneidensis* MR-1, substrate type: sodium lactate, the volume of each chamber: 30 mL, cathodic pH: 3, catholyte: (100 μM TPTC, 2% NaCL), air flow rate: 100 mL min^−1^, temperature: 30 °C	57.25 mW m^−2^	68
Bisphenol A, estrone, sulfamethazine, triclocarban	PEM	Graphite rod with graphite granules	Graphite rod with graphite granules	—	In batch mode: 90–100% E1, 56–100% SM2, 58–99%TCC, 65–73% BPA	24 h	ER: 10 Ω, anodic inoculum: anaerobic sludge, substrate type: glucose/acetate, the volume of each chamber: 125 mL, cathodic pH: 3, catholyte: (1 mg L^−1^ of each EC, 0.1 M Na_2_SO_4_ and 1.25 mM FeSO_4_)	0.29–1.11 w m^−3^	93
					In continuous mode: 94–100% E1, 62–97% SM2, 62–98% TCC, 64–75% BPA				
Carbamazepine	—	Carbon brush	Carbon cloth with gas diffusion cathode (GDC)	—	90 ± 3%	24 h	ER: 10 Ω, substrate type: acetate, the working volume of cell: 28 mL, catholyte: (10 mg L^−1^ CBZ and 5 g L^−1^ Na_2_SO_4_), iron catalyst: Fe–Mn binary oxide, temperature: 30 ± 3 °C	112 ± 11 mW m^−2^	62
NSAIDs (ibuprofen, diclofenac, ketoprofen, naproxen)	Bipolar membrane	Carbon brush	Graphite plate and Ag/AgCl reference electrode	—	80–86% ibuprofen, 87–97% diclofenac, 59–61% ketoprofen, 75–81% naproxen	5 h	ER: 0.1 Ω, anodic inoculum: domestic wastewater, substrate type: sodium acetate, total volume of each chamber: 100 mL, cathodic pH: 2, catholyte: (40 μg L^−1^ of each NSAID, 0.05 M Na_2_SO_4_ and 7.5 mM FeSO_4_), applied voltage: 0.3 V, air flow rate: 8 mL min^−1^, temperature: 25 ± 5 °C	—	146
Erythromycin (ERY) and antibiotic resistant genes (ARGs)	CEM	Carbon cloth	CNT/γ-FeOOH/stainless steel mesh	88.73% ERY	100% erm B, 77.6% erm C, 63.5% ermG	48 h	ER: 1000 Ω, anodic inoculum: effluent from another MFC and electrolyte, substrate (electron donor): ERY wastewater (50 μg L^−1^), the volume of each chamber: 76.3 mL, cathodic pH: 7 (with PBS), catholyte: (Anode effluent containing ARGs), air flow rate: 100 mL min^−1^, temperature: 30 °C	0.193 W m^−2^	57
Tylosin, sulfaquinoxaline, tetracycline	CEM	Carbon felt	Carbon felt	—	85.9–88.2% antibiotics	17 h	ER: 1000 Ω, anodic inoculum: anaerobic sludge, substrate type: sodium acetate, the volume of each chamber: 250 mL, cathodic pH: 3, catholyte: (10 mg L^−1^ of each antibiotic), iron catalyst: SrM-NPs (0.3 g L^−1^), air flow rate: 100 mL^−1^, temperature: 24 ± 2 °C	136.4 ± 3.1 mW m^−2^	73
Sulfamethoxazole	PEM (Nafion)	Carbon cloth	CNT/γ-FeOOH/stainless steel mesh	94.66%	—	48 h	ER: 1000 Ω, anodic inoculum: effluent from another MFCs, substrate (electron donor): SMX wastewater (25 mg L^−1^), the volume of each chamber: 28 mL, cathodic pH: 7 (with PBS), air flow rate: 20 mL min^−1^, temperature: 30 °C	283.32 ± 16.35 mW m^−2^	71
Tetracycline	PEM (Nafion)	Carbon felt	Carbon felt	85.71 ± 1.81%	COD 99.04 ± 0.91%	24 h	Short-circuit condition, anodic inoculum: anaerobic sludge, substrate type: glucose, the volume of each chamber: 450 mL, cathodic pH: 3, catholyte: (10 mg L^−1^ of tetracycline, 0.1 M Na_2_SO_4_ and 5 mg L^−1^ FeSO_4_), temperature: 30 °C	141.60 mW m^−2^	81

The advanced removal of ECs including bisphenol A (BPA), estrone (E1), sulfamethazine (SM2), and triclocarban (TCC) examined by Wang *et al.* using the BEF system. E1, SM2, TCC and BPA removal efficiencies in MFC batch mode for 24 h were 90–100%, 56–100%, 58–99%, and 65–73%, respectively. On the other hand, the removal efficiencies in the continuous mode were 94–100%, 62–97%, 62–98%, and 64–75%, respectively. The use of glucose and graphite rods along with graphite granules as a substrate and electrode, respectively, increased the current density in the MFC system and the H_2_O_2_ production at the cathode. The absorption on the graphite electrode and the oxidation by ˙OH through the Fenton's reaction resulted in the contaminants removal.^93^ Xu *et al.* reported a similar case when using BEF system equipped with Fe@Fe_2_O_3_/NCF cathode electrode for removing steroid hormones such as 17β-estradiol (E2) and 17α-ethynyl-estradiol (EE2) as the most potent EDCs.^33^ The results showed that the removal mechanisms of E2 and EE2 were absorption and oxidation. Generally, steroid hormones were adsorbed on the electrode. Hydroxyl free radicals were generated from the Fenton's reaction between *in situ* electrogenerated H_2_O_2_ and ferrous ions leached from the Fe@Fe_2_O_3_/NCF under acidic pH in order to E2 and EE2 oxidation. However, zero-valent iron particles were possibly reacted with O_2_ to form reactive intermediates (*e.g.*, ˙OH, HO_2_/˙O_2_, and H_2_O_2_). Thus, the absorption and oxidation mechanisms in the cathode chamber, resulted in the removal of 81% of E2 and 56% of EE2 under closed-circuit condition during 10 h. Two intermediates of 6-OH-E2 and E1 were detected with GC/MS during the E2 oxidation. The total iron ion concentration was reached 1.21 mg L^−1^ within 10 h in the BEF system equipped with Fe@Fe_2_O_3_/NCF under closed-circuit condition. Furthermore, the maximum power density and the steady current were 4.35 W m^−3^ and 0.60 mA, respectively. In similar another study, Xu and co-workers also achieved the removal of E2 using a BEF system equipped with two Fe@Fe_2_O_3_/NCF electrodes. Over 90% of E2 was removed after 6 h when the external resistance was close to the internal. Oxidation mechanism played an important role in removal of E2 instead of cathodic sorption in BEF system.^148^

Among ECs, PPCPs with low concentrations and long-term hazards may enter the aquatic environment and cause potential problems due to their high consumption and incomplete removal by conventional treatment methods in municipal wastewater treatment plants.^144,145,149^ Carbamazepine (CBZ) as an anti-epileptic drug is one of the most commonly identified drugs in wastewater.^150^ The removal of CBZ using a BEF system, a combination of EF system and single-chamber MFC, was performed by selecting Fe–Mn binary oxide as the Fenton catalyst to produce ˙OH in a study by Wang *et al.*^62^ The maximum power density of 112 ± 11 mW m^−2^ was recorded using acetate as a substrate. The performance of the BEF system was attributed to the synergistic mechanisms in the anode and cathode chamber. The acetate substrate was utilized to release the electrons and protons in the anode, and the H_2_O_2_ and ferrous iron were reacted to produce the ˙OH. The synergistic reactions between CBZ oxidized by ˙OH and intermediates biodegraded by microorganisms, resulted in the advanced degradation of 90% of CBZ in 24 h. Hydroxycarbamazepine was recognized as one of the primary intermediates during the CBZ oxidation with ˙OH production. The ˙OH oxidized CBZ intermediates to form acridone. This study reported that acridone intermediate can be biodegraded as the substrate by microorganisms and converted to simpler oxidation products (CO_2_ and H_2_O). In contrast, Nadais *et al.*^146^ investigated the efficiency of the two-chamber BEF system based on microbial electrolysis cell for analyzing the degradation of four Non-Steroidal Anti-Inflammatory Drugs (NSAIDs) in wastewater. The parameter values of Fe^2+^ = 7.5 mM, pH = 2, applied voltage = 0.3 V, and air flow rate = 8 mL min^−1^ were reported as optimal conditions. During the 5 h reaction time, the removal efficiencies of diclofenac, ketoprofen, naproxen, and ibuprofen were 87–97%, 59–61%, 75–81%, and 80–86%, respectively. Moreover, the BEF process was introduced as a suitable alternative to wastewater treatment with low concentrations of environmental organic pollutants. In another study, Li *et al.* also used a two-chamber BEF process to erythromycin (ERY) removal. The results showed that 88.73% of ERY in the anode chamber, 100% erm B, 77.6% erm C, and 63.5% erm G as antibiotic-resistant genes (ARGs) in the cathode chamber of BEF system equipped with γ-FeOOH/CNT/stainless-steel-mesh composite electrode were decomposed under neutral pH in 48 h. To prepare a composite electrode, γ-FeOOH as Heterogeneous Fenton catalyst was prepared with 4 g of FeCl_2_·4H_2_O, 7 g of (CH_2_)_6_N_4_ and 1.75 g of NaNO_2_ and were dissolved in 80, 20 and 20 mL of distilled water, respectively. Then, the three solutions were mixed to form a bluish green precipitate and the precipitate was aged at 65 °C for 3 h. In the next step, after centrifugation of the entire precipitate, 95% alcohol was utilized to wash it three time successively. Also, the washing was repeated three time with distilled water to remove impurities and the mixture was dried at 65 °C for 48 h. Then, 5 g of γ-FeOOH and 5 g of CNT were mixed with 0.5 g of polytetrafluoroethylene (PTFE) and ethanol in an ultrasonic bath and the dough-like paste was assembled into between two pieces of Ti mesh. The reason of the relatively high degradation of ERY was the metabolism of anodic microorganisms. Moreover, the Fenton oxidation reactions with the ˙OH production resulted in the ARGs degradation. The maximum power density 0.193 W m^−2^ was obtained by adding a low ERY concentration at the anode.^57^ Additionally, Li *et al.*^71^ also utilized similar CNT/γ-FeOOH/stainless steel mesh cathodic electrode to analyzed sulfamethoxazole (SMX) degradation in a two-chambered BEF system. An optimal concentration of 25 mg L^−1^ resulted in a high removal (94.66%) of SMX during 48 h. To determine the mechanism of SMX mineralization, GC-MS was utilized to identify intermediates. The proposed degradation pathway by BEF was that SMX added a C atom and transformed it to sulfamoxole. In the next step, SMX was converted into 3-amino-5 methyl-isoxazole and 4-aminobenzenesulfinic acid. Afterward, 3-amino-5-methyl-isoxazole was converted into 5-aminoisoxazole and also 4-aminobenzenesulfinic acid was transformed into phenol or aniline. Detection of these intermediates had shown that SMX was hydrolyzed. In this study, the intermediates of SMX degradation were produced *via* hydroxylation and acetylation reactions. Moreover, a maximum power density of 283.32 ± 16.35 mW m^−2^ was obtained. This issue highlighted the efficiency of BEF regarding the effective treatment of SMX effluent. Similarly, Wang *et al.*^69^ used γ-FeOOH as a heterogeneous iron catalyst. In their study, a two-chambered BEF system was equipped with a γ-FeOOH/CF composite cathode electrode to remove and oxidize As(iii). The γ-FeOOH/CF composite electrode was prepared *via* mixing polyvinylidene fluoride (PVDF) and FeOOH with a solution of 1-methyl-2-pyrrolidone in an ultrasonic bath in order to form a dough-like paste. Finally, using a pressure of 10 MPa, the paste was assembled into the CF at 60 °C and keeping this temperature for 24 h. As(iii) was rapidly oxidized to As(v) with lower toxicity by ˙OH radicals at the cathode chamber. The increase in the γ-FeOOH dose resulted in the increased As(v) adsorption on the active sites of the iron surface. Approximately 96% of As(iii) was removed during 72 h in BEF process, which contained 2 g of γ-FeOOH, and the concentration of *in situ* produced Fe^2+^ at the end of the reaction time was 0.57 mg L^−1^. In addition, the maximum power density was determined to be 135.3 mW m^−2^.

In another study, Hassan *et al.*^73^ developed M-type strontium hexaferrite magnetic nanoparticles (SrM-NPs) as a novel heterogeneous Fenton catalyst and the removal of three antibiotics, including tylosin, sulfaquinoxaline, and tetracycline examined in a two-chamber BEF system. The chemical co-precipitation method was used to preparation of SrM-NPs. The chemicals of strontium nitrate, iron chloride and sodium hydroxide were served. These chemicals were dissolved in deionized water at 70 °C under stirring. In addition, the aqueous solutions were stirred for 2 h in order to homogenize the hexaferrite solutions. Then, the suspensions were filtered and also washed several times to get a neutral pH. In order to obtain a fine magnetic powder, the filtered residues were dried at 150 °C and then ground for 30 min. Finally, the powder was calcined at 1000 °C for 3 h. The removal of 85.9–88.2% of tylosin, sulfaquinoxaline, and tetracycline was achieved during 17 h under optimal conditions (SrM dose = 0.3 g L^−1^, pH = 3) with the ˙OH production as a strong oxidant. Moreover, their complete degradation with removing 74.8–87.2% of total organic carbon (TOC) was achieved during 24 h. The researchers reported that the antibiotics degradation by SrM-NPs (Fenton heterogeneous catalysts) was higher (approximately 100%) than the antibiotics degradation by Fenton homogeneous catalysts (FeSO_4_). In addition to the high removal of antibiotics by the SrM heterogeneous catalyst, a small amount of iron ions was remained in the solution at the end of the process. Therefore, the amount of residual iron was reported in the range of 0.12 to 0.23 mg L^−1^ at catalyst concentrations of 0.1–0.5 g L^−1^, which was lesser than the iron content generated at 0.3 g L^−1^ FeSO_4_ (1.74 mg L^−1^). In a study conducted by Tong *et al.*^68^ the degradation of Triphenyltin chloride (TPTC) evaluated in the BEF process equipped with cathodic electrode composition of Fe@Fe_2_O_3_/graphite felt. The degradation efficiency of TPTC under cathodic Fenton's reactions reached 78.32 ± 2.07% in 101 h with 100 μmol L^−1^ as the optimal concentration of TPTC. The investigation results of the TPTC degradation mechanism were shown that TPTC removal initially involved *via* breaking down tin-carbon bonds, and TPTC was degraded by the attack of ˙OH on phenyl group to create an adduct between ˙OH and the benzene ring. This study reported that TPTC was degraded to diphenyltin (DPT) and monophenyltin (MPT) products and the inorganic tin and CO_2_ were formed at the end of the process. In addition, the current production in MFC was used to produce H_2_O_2_ up to 135.96 μmol L^−1^ at the cathode, and the maximum power density was 57.25 mW m^−2^ at current density of 252.22 mA m^−2^.

*P*-Nitrophenol (PNP) in wastewater is a raw material in the chemical industries and a priority toxic pollutant.^151,152^ Zhu and Ni applied the BEF process to remove *p*-nitrophenol, and as well as to produce energy simultaneously. The complete degradation of PNP was achieved after 12 h. In addition, the removal of 85% of TOC was attained after 96 h due to the reaction H_2_O_2_ with Fe^2+^ released from scrap iron as an iron source along with ˙OH formation. Simultaneously, 143 mW m^−2^ was determined to be the maximum power density.^58^ In another study,^106^ the removal efficiency of PNP using natural limonite as iron catalyst was 96% during 6 h in BEF system. Moreover, in a study conducted by Zhu *et al.*, the BEF system with the single-chamber MFC was used as an energy source to phenol degradation. The results showed that under acidic conditions, phenol was completely decomposed into simple organic acids after 22 h, and 75% of TOC was removed. In general, phenol decomposition was performed in three steps. In first step, aromatic intermediates (*e.g.*, hydroquinone) were produced through radical reactions by ˙OH. Then, aromatic intermediates were decomposed with ring breakage to different carboxylic acids (*i.e.*, maleic acid, fumaric acid, formic acid and oxalic acid). Eventually, carboxylic acids were mineralized to CO_2_.^147^

### 3.2. Dyes

The color substances in water are the result of the presence of natural dyes and the entry of industrial color-contaminated wastewaters into the water bodies.^153^ Synthetic dyes are most common contaminants in industrial wastewaters.^88,154^ The colored wastewaters of textile industry are full of dangerous organic dyes and result in important environmental and ecological problems.^22,155,156^ The widespread discharge of the color-contaminated industrial wastewater into water sources causes serious problems such as negative impacts on the aesthetic aspects, the disruption of the photosynthetic activity in aquatic environments, preventing the transfer of sunlight and oxygen to the water. Moreover, the residual dyes in water bodies can cause harmful effects on aquatic life due to their toxic and carcinogenic properties. The presence of dyes in the environment also results in various health problems, including allergies, skin irritations, cancers, and genetic mutations in humans.^155,157–162^

Azo dyes have the highest production rate (70%) worldwide. The azo dyes such as Alizarin red S, Evans blue, Amaranth, Congo red, *etc.* have been used for coloring the different products.^22,65,161^ Azo dyes (with the –Ne=Ne– group) have complex and stable structures due to the presence of euxochromes and chromophores. These structures complicate the process of azo dye degradation using conventional methods.^155,157,163,164^ Thus, the effective decolorization and detoxification of colored effluents is one of the environmental regulations.^165,166^

In recent years, scientists have focused on the BEF systems to degrade colored pollutants and to prevent their negative effects on the environment.^35,67,90^ As shown in Table 4, the degradation of some industrial dyes such as rhodamine B,^63^ amaranth,^65^ orange II,^35,140^ methyl orange,^109^ acid orange 7,^167^*etc.* has been reported by the BEF systems. Zhuang *et al.*^63^ examined the application of a BEF system with a CF anode electrode, three different types of cathode electrodes of (1) NCF, (2) Fe^2+^/NCF, (3) Fe@Fe_2_O_3_/NCF, and brewery wastewater as the anode inoculum for rhodamine B dye (Rh B) degradation in the cathode chamber. The power densities with using NCF, Fe^2+^/NCF, Fe@Fe_2_O_3_/NCF were 56, 142 and 307 mW m^−2^, respectively. Based on the dependence of Rh B mineralization and decolorization to cathodic current density (in short-circuit conditions with increasing current density), the rate of Rh B removal reached 95% using a Fe@Fe_2_O_3_/NCF composite cathodic electrode in 12 h. Moreover, 90% of the TOC was eliminated under these conditions. In another study,^74^ 95% of Rh B was also removed by applying the optimal conditions of cathodic pH of 3, external resistance of 120 Ω, and air flow of 0.3 L min^−1^ in a two-chamber BEF system equipped with the Fe@Fe_2_O_3_/ACF electrode cathode along with the production of a maximum power density of 16.7 W m^−3^. The investigation of the Rh B degradation mechanism was shown that the Hydroxyl radicals attacked the Rh B structure at first and opened the chromogenic group of Rh B. Some major intermediates were generated, including *o*-phthalic acid, benzoic acid, benzyloxyamine, 2-hydroxyglutaric acid and 2-hydroxybenzoic acid. Then, *o*-phthalic acid was transformed into *p*-phthalic acid and *m*-phthalic acid or was converted into *o*-phthalic anhydride *via* the dehydration condensation reaction. Also, 2-hydroxyglutaric acid was mineralized to CO_2_ and H_2_O. Afterward, all molecular substances with benzene ring were decomposed to malonic acid, succinic acid, ethylene glycol and oxalic acid. Small substances were also mineralized into CO_2_ and H_2_O.

**Table 4:** Literature review on application of BEF systems for dye removal

Target dye type	Membrane	Anodic electrode	Cathodic electrode	Removal efficiency		Operation time	Operation conditions	Max. power generation	Ref.
				Anode	Cathode				
Rhodamine B (Rh B)	GORE-TEX® cloth	Carbon felt	NCF, Fe^2+^/NCF, Fe@Fe_2_O_3_/NCF	—	95% with Fe@Fe_2_O_3_/NCF	12 h	ER: 1000 Ω, anodic inoculum: microbial community of another MFC, the volume of each chamber: 75 mL, cathodic pH: 3 (HCL), catholyte: (Artificial dye wastewater with 15 mg L^−1^ Rh B), temperature: 30 °C	307 mW m^−2^	63
Amaranth	PEM (Nafion-112)	Graphite granules	Spectrographic pure graphite rod	—	76.43–82.59%	1 h	ER: 20 Ω, substrate type: glucose, the volume of each chamber: 80 mL, cathodic pH: 3, catholyte: (75 mg L^−1^ amaranth, 0.1 M Na_2_SO_4_, FeSO_4_ concentration: 1.0 and 0.5 mM), temperature: 20 °C	28.3 W m^−3^	65
Orange II	CEM	Carbon felt	CNT/PTFE/γ-FeOOH	—	100% (complete decolorization) (complete mineralization)	14 h, 43 h	ER: 1000 Ω, anodic inoculum: Shewanella decolorationis S12, substrate type: lactate, the volume of each chamber: 75.6 mL, cathodic pH: 7, catholyte: (0.1 mM orange II, 100 mM PBS), air flow rate: 100 mL min^−1^, temperature: 30 °C	230 mW m^−2^	35
Azo dye (orange II)	CEM	PPy/AQDS modified carbon felt	PPy/AQDS modified carbon felt	—	*K*_decolori*z*ation_ = 0.142–0.358 h^−1^	20 h	ER: 1000 Ω, anodic inoculum: Shewanella decolorationis S12, substrate type: lactate, the volume of each chamber: 75 mL, cathodic pH: 7, catholyte: (0.2 mM orange II, 0.1 M PBS and 1 g L^−1^ γ-FeOOH powder), air flow rate: 100 mL min^−1^, temperature: 30 °C	823 mW cm^−2^	140
Acid orange 7 (AO7)	PEM (Nafion-212)	Carbon cloth	Carbon felt	100% furfural	89% AO7	60 h	ER: 1000 Ω, anodic inoculum: anaerobic sludge, substrate type: glucose and 300 mg L^−1^ furfural, the volume of each chamber: 100 mL, cathodic pH: 3, catholyte: (50 mg L^−1^ AO7, 20 g L^−1^ Na_2_SO_4_ and 1 g FeVO_4_ (iron catalyst), temperature: 30 ± 1 °C	15.9 W m^−3^	169
Acid orange 7	PEM	Iron plate	Carbon paper	85% AO7	—	30 min	ER: 1000 Ω, anodic inoculum: anaerobic sludge, substrate type: sodium acetate, the volume of each chamber: 200 mL, cathodic pH of MFC: 7 (PBS), anodic pH: 3, anolyte of MFC: (0.1 to 2 mM AO7, 0.16 M NaCl), catholyte of MFC: 0.16 M NaCl	0.3 mW	167
Lissamine green B, indigo carmine, crystal violet, reactive black 5, poly R-478	Salt bridge	Graphite sheet	Graphite sheet	—	98.2% LG, 97.2% IC, 96.2% CV, 88.2% RB5 19.1% poly R-478	15 min, 60 min	ER: 1000 Ω, anodic inoculum: sewage sludge, substrate type: marine sediment with acetate, the working volume of cathode chamber: 0.15 L, catholyte: (150 mL dye solution, 0.01 M Na_2_SO_4_ and iron concentration: 150 mg L^−1^), air flow rate: 1.0 L min^−1^	1033–1046 mV	64
Methylene blue (MB)	Bipolar membrane	Carbon fiber brush	Graphite plate and Pt coated carbon paper	—	97% (decolorization)	8 h	ER: 5 Ω, anodic inoculum: domestic wastewater, substrate type: acetate, the working volume of each chamber: 250 mL, cathodic pH: 3, catholyte: (50 mg L^−1^ MB, 0.1 M Na_2_SO_4_ and iron concentration: 2 mM), air flow rate: 10 mL min^−1^, temperature: 25 ± 5 °C	50.1 mW m^−2^	98
				99.6%(mineralization)	16 h				
Methyl orange (MO)	CEM	Graphite fiber brush	Fe_2_O_3_/active carbon felt	—	73.9–86.7%	2 h	ER: 100 Ω, anodic inoculum: anaerobic sludge, the working volume of each chamber: 550 mL, cathodic pH: 3, catholyte: (5 mg L^−1^ MO and 0.05 M Na_2_SO_4_), air flow rate: 750 mL min^−1^	268.10 mW m^−3^	109
Congo red	PEM (Nafion-117)	Graphite rod with graphite granules	Anthraquinone-based spherical catalyst and graphite rod	—	90%	72 h	ER: 1000 Ω, anodic inoculum: microbial community of an another MFC, substrate type: glucose, the volume of each chamber: 100 mL, cathodic pH: 7, catholyte: (100 μM Congo red and 0.1 M PBS), air flow rate: 8 ± 0.5 mg L^−1^, temperature: 20 ± 3 °C	808.3 mW m^−3^	168
Rhodamine B	CEM	Active carbon felt	Fe@Fe_2_O_3_/active carbon felt	85.2 ± 3.3% BOD_5_, 75.1 ± 3.1% COD, Rh B 60.5 ± 2.8% TOC of swine wastewater	95.0 ± 3.5% of Rh B	24 h	ER: 120 Ω, anodic inoculum: anaerobic sludge, substrate type: lactate, the volume of two chambers: 200 mL, anodic pH: 10, cathodic pH: 3, anolyte: swine wastewater, catholyte: (10 mg L^−1^ Rh B, 0.1 M NaCL), air flow rate: 0.3 L min^−1^, temperature: 25 °C	16.7 W m^−3^	74

Fu *et al.*^65^ focused on the amaranth degradation in the MFC-Fenton system at pH 3, which is a stable and resistant azo dye to degradation by H_2_O_2_. The MFC-conventional Fenton system removed 82.59% of the amaranth in 1 h when the optimum concentration of 1 mmol L^−1^ of ferrous iron was added to the cathode. Moreover, this dye was degraded in the MFC-electrochemical Fenton system with 0.5 mmol L^−1^ of ferric ions as Fenton catalyst, at the removal rate of 76.4% by ˙OH. In addition, the maximum power density was 28.3 W m^−3^. While the complete decolorization and mineralization of Orange II achieved in the study of Feng *et al.* at pH 7 during 14 and 43 h, respectively, in the BEF process. A CF anode electrode, a γ-FeOOH/PTFE/CNT electrode as a cathode electrode, and Shewanella decolorationis S12 as an active biocatalyst in the anaerobic anode chamber of a two-chambered MFC reactor used. When the ratio of CNT to γ-FeOOH was 1 : 1, Orange II was degraded rapidly, and the highest amount of H_2_O_2_ was produced due to the effect of electrode cathodic composition on the performance of the BEF system. In addition, the concentration of *in situ* produced Fe^2+^ was 1.62 mg L^−1^ after a 50 h reaction. The maximum power density of 230 mW m^−2^ was also obtained simultaneously.^35^ Another similar study reported that BEF is an environmentally-friendly system capable of degrading azo dyes effectively. Based on this point, Ling *et al.* have conducted a research and came out with the conclusion that methyl orange (MO) was effectively degraded during eight batch operations in BEF system with graphite fiber brush and Fe_2_O_3_/ACF as anodic and cathodic electrode, respectively. The oxidative degradation efficiency of this dye varied from 73.9% to 86.7% and the amount of H_2_O_2_ generation reached 88.63 μmol L^−1^ under the optimal conditions of this system.^109^

To solve the problem of low power generation in benthonic MFC (BMFC), a research team^64^ used a new hybrid system, which integrates BMFC anode with an EF cathode in order to: (1) remove different dyes; and (2) produce sustainable energy. The anode was buried in organic-rich material (including marine sediments, sludge, and a mixture of both) and connected by a salt bridge to the EF cathode chamber where the EF reactions take place. The degradation rate of Lissamine Green B, Indigo Carmine, Crystal Violet, and Reactive Black 5 were 98.2%, 97.2%, 96.2%, and 88.2%, respectively in 15 minutes. Furthermore, the rate of poly R-478 decolourisation was 19.1% in 1 h due to EF reactions in the cathode chamber of this hybrid system. The results clearly showed that the EF and MFC hybrid system is a stable and cost-effective system for decolourizing colored wastewaters. In another similar experiment, the integrated BEF system, which is the result of the integration of the MFC reactor and the catalytic oxidation reactor (COR), was utilized for degrading the Congo red.^168^ The dissolved oxygen and the produced H_2_O_2_ as desirable oxidants led to Congo red degradation in the COR reactor due to the iron phthalocyanine catalyst (FePc). Consequently, under neutral conditions, more than 90% of this dye (with a maximum power density of 808.3 mW m^−3^) was degraded during 72 h. Six types of residual organic acids were identified as the degradation products of Congo red, including 2-(carboxy(hydroxy)methyl)benzoic acid, malonic acid, maleic acid, 5-oxo-4,5-dihydrofuran-3-carboxylic acid, benzene-1,2,4,5-tetraol and 4-hydroxynaphthalene-1-sulfonic acid. It showed that cyclic structures and azo bonds of Congo red could be eliminated in the MFC-COR system.

The refractory organic pollutants degradation in the anode and cathode chambers of MFC combined with a Fenton-like system as a BEF system was performed simultaneously with the production of a maximum power density of 15.9 W m^−3^ in a study conducted by Luo *et al.* After the removal of 100% of the furfural contaminant in the anode chamber, the Fenton's reactions were performed using FeVO_4_ catalyst as a heterogeneous iron source. FeVO_4_ catalyst was prepared *via* a wet chemical method. Under a 1 : 1 molar ratio, a 0.26 M iron nitrate solution was rapidly poured into a 4.27 × 10^−2^ M ammonium metavanadate solution in the stirring condition and the mixture was kept at 75 °C for 1 h. Then, the precipitate was separated *via* pumping filtration, and washed with acetone and ultrapure water. Eventually, the precipitate was dried in an oven at 50 °C for about 15 h. The removal of 89% of Acid Orange 7 (AO7) and 81% of COD in the cathode chamber under the optimal pH of 3 and FeVO_4_ powder of 0.8 g were obtained. Generally, the furfural was biodegraded by microorganisms to produce electrons and protons in the anode chamber. Then, after transferring electrons and protons to the cathode chamber, AO7 was possibly reduced through two reactions such as the Fenton-like reactions and the electrochemical reductions. In the electrochemical reduction mechanism, the azo bonds were broken *via* protons and electrons and products of 1-amino-2-naphthol and sulfanilic acid were created. However, Fenton-like reactions with the ˙OH production, which was generated from the reaction of H_2_O_2_ and FeVO_4_, resulted in maximum degradation of AO7. GC/MS results were shown some intermediates, such as naphthalene, benzaldehyde, and phenol. Also, further oxidation of intermediates led to the mineralization into CO_2_ and H_2_O.^169^ In contrast to the cathodic removal of AO7, Liu *et al.* developed an innovative anodic Fenton treatment (AFT) system combined with the air cathode MFC to treat organic pollutants. The degradation rate of AO7 in integrated system was higher than the chemical Fenton processes. Moreover, it was found that the increase in the cathodic solution oxygen of the MFC system, which led to an increase in the power density, took place along with the increase in the degradation rate of AO7. That is, about 85% of AO7 was degraded at pH of 3 and with the addition of 2 mM H_2_O_2_ with a power output of 0.3 mW.^167^

Residual H_2_O_2_ after Fenton treatment may cause errors in measuring chemical oxygen demand (COD) and biochemical oxygen demand (BOD). Hence, an ideal technology is needed to remove the residual H_2_O_2_ from the Fenton process. To solve this problem, Zhang *et al.* have developed an innovative BEF system that was able to alternately switch between MEC and MFC operation to degrade Methylene blue (MB) dye and to control H_2_O_2_ concentration. In this system, after the generation of a maximum current density of 0.49 A m^−2^, the residual H_2_O_2_ of 180 mg L^−1^ was completely removed by switching from MEC to MFC during 36 h. In the MEC mode, MB decolorization and mineralization efficiencies were 97% in 8 h and 99.6% in 16 h, respectively. Electrode adsorption, Fenton-based reactions, and H_2_O_2_ production were important reasons behind the degradation of MB. Therefore, the treatment of colored wastewaters due to high pollution load and treatment problems has become one of the most challenging issues in water and wastewater treatment and has attracted the researchers' attention.^98^

### 3.3. Real wastewaters

The entry of wastewater into the environment without proper treatment leads to serious health and environmental problems due to its toxic and hazardous organic compounds.^170,171^ Therefore, despite these problems, one of the most important environmental challenges is the presence of persistent organic pollutants in wastewater, which are not easily removed using conventional methods.^172^ In order to prevent the negative effects of wastewater on the environment, it must be treated in accordance with the environmental discharge standards.^171,173^ The BEF technologies are emerging and promising options for treating real wastewater and show high organic matter removal.^57^ According to the Table 5, the application of BEF process for the treatment of different types of real wastewaters has been investigated.

**Table 5:** Literature review on application of BEF systems for real wastewaters treatment

Target wastewater	Membrane	Anodic electrode	Cathodic electrode	Removal efficiency		Operation time	Operation conditions	Max. power generation	Ref.
				Anode	Cathode				
Real landfill leachate	CEM	Carbon felt	Natural pyrrhotite-coated graphite	—	78% COD, 77% color	45 d	ER: 500 Ω, anodic inoculum: anaerobic sludge, substrate type: sodium acetate, the working volume of two chambers: 850 mL, cathodic pH: 5.4, catholyte: (Landfill leachate solution with 1022 mg L^−1^ COD, 1 M KCl)	4.2 W m^−3^	181
Brewery wastewater	GORE-TEX® cloth	Carbon felt	Fe@Fe_2_O_3_/NCF	—	—	—	ER: 1000 Ω, anodic inoculum: microbial community of an another MFC, substrate type: brewery waste water, the volume of each chamber: 75 mL, cathodic pH: 3, catholyte: (2% NaCl and fenton's reagents), air flow rate: 300 mL min^−1^, temperature: 30 °C	341.4 mW m^−2^	86
Swine wastewater	GORE-TEX® cloth	Graphite granules with graphite rod	Fe@Fe_2_O_3_/carbon felt	38.9–46.5% COD, 62.3–71.7% BOD_5_, 35.1–38.4% TOC, 53.5–58.7% NH_3_–N	23.3–30.2% COD, 20.6–25.5% BOD_5_, 46.2–57.3% TOC, 23.3–29.8% NH_3_–N	12 d	ER: 100 Ω, anodic inoculum: brewery waste water, substrate types: glucose, sucrose, acetate and swine wastewater, the volume of two chambers: 800 mL, cathodic pH: 3, catholyte: (swine wastewater), air flow rate: 300 mL min^−1^, temperature: 30 °C	3.1–7.9 mW m^−3^	61
Oily wastewater	PEM (Nafion-117)	Carbon felt	Carbon felt	—	40% COD	4 h	ER: 1000 Ω, anodic inoculum: dairy anaerobic sludge, substrate types: dairy wastewater, the volume of two chambers: 1500 mL, cathodic pH: 3, catholyte: (oily wastewater, 0.75 mM FeSO_4_)	102 mW m^−2^	176
Medicinal herbs wastewater	PEM (Nafion-112)	Graphite plate	Fe@Fe_2_O_3_/graphite	78.05% COD	84.02% COD	50 h	ER: 100 Ω, anodic inoculum: anaerobic sludge, substrate type: medicinal herbs wastewater, the working volume of each chamber: 450 mL, cathodic pH: 3, catholyte: (medicinal herbs wastewater), air flow rate: 300 mL min^−1^, temperature: 30 °C	49.76 mW m^−2^	78
Aniline wastewater	Bipolar membrane	Carbon fiber brush	Graphite plate	—	97.1 ± 1.2%	6 d	Anodic inoculum: domestic wastewater, substrate type: sodium acetate, the working volume of two chambers: 100 mL, cathodic pH: 3, catholyte: (4460 ± 52 mg L^−1^ aniline, 50 mM Na_2_SO_4_ and FeSO_4_ concentration: 10 mM), air flow rate: 16 mL min^−1^, applied voltage: 0.5 V, temperature: 20 ± 2 °C	—	177
Real landfill leachate	CEM	Carbon felt	Carbon felt	49.3 ± 6.5% COD	40.7 ± 3.1% COD	83 d	Anodic inoculum: sludge from another MFC, substrate types: glucose and pre-treated landfill leachate, the working volume of each chamber: 280 mL, cathodic pH: 3, catholyte: (Synthetic and real leachate, 300 mg L^−1^ FeSO_4_), temperature: 25 ± 1 °C	1.7 A m^−2^	99
Real landfill leachate	CEM	Granular activated carbon along with graphite granules embedded between SS wire meshes and graphite rod	Stainless steel (SS) wire mesh and SS rod	In batch mode: 318–351 mg L^−1^ d^−1^ COD, in continuous mode: 1077–1244 mg L^−1^d^−1^ COD	In batch mode: 254–289 mg L^−1^d^−1^ COD, in continuous mode: 863–897 mg L^−1^d^−1^ COD	35 d	Anodic inoculum: effluent from another MFC, substrate types: glucose and pre-treated landfill leachate, the working volume of two chambers: 1100 mL, cathodic pH: 3, catholyte: (effluent of pretreated leachate, iron concentration: 300 mg L^−1^ FeSO_4_), temperature: 25 ± 4 °C	43.5 ± 2.1 A m^−3^	178
Coal gasification wastewater (CGW)	PEM (Nafion-212)	Carbon felt	FeVO_4_/carbon felt	—	83.7% COD, 92.3% BOD_5_, 91.5% TOC, 85.7% total phenols	—	ER: 118.6 Ω, anodic inoculum: microbial community of a MFC, substrate types: glucose, the volume of two chambers: 200 mL, cathodic pH: 7, catholyte: (100 mL CGW, 0.1 M Na_2_SO_4_), air flow rate: 100 mL min^−1^, temperature: 30 °C	849.7 mW m^−3^	100
Real landfill leachate	—	—	nZVI@ Modified activated carbon (MAC)	—	72.2–83.8%	30 h	ER: 1000 Ω, inoculum of single chamber MFC: nutrients buffer solution, substrate type: leachate, the working volume of two chambers: 28 mL, iron source: nano-zero-valent iron, temperature: 25 °C	0.9–1.0 W m^−2^	179

Swine wastewater, as a complex type of wastewater, has a large amount of organic matter, nitrogen, and phosphorus. The presence of these substances in water sources causes serious pollution.^174,175^ Xu *et al.*^61^ used a BES system with cathodic BEF and anodic oxidation without an external energy source in order to treat swine wastewater. The BES reactor was used with two cylindrical chambers separated *via* GORE-TEX, the outer anode chamber with a graphite rod and filled with the granular graphite, and the internal cathode chamber with five Fe@Fe_2_O_3_/CF electrodes. The performance of the system was evaluated based on two different organic loading rates (OLRs) of 1.1 g COD L^−1^ d^−1^ and 6.4 g COD L^−1^ d^−1^. The removal efficiencies of COD, TOC, BOD_5_, and NH_3_–N in different OLRs were reported to range from 62% to 95%. The organic contaminants in the anode chamber were not completely removed by the oxidation reactions of microorganisms. A number of them were presented in the anode, and the anode effluent was continuously pumped into the cathode for further treatment. In addition to the anodic removal of carbonaceous contaminants, the swine effluent contaminants were oxidized at the cathode due to the presence of strong ˙OH. Moreover, the maximum power density in OLR of 1.1 g COD L^−1^ d^−1^ and 6.4 g COD L^−1^ d^−1^, were 3.1 and 7.9 mW m^−3^, respectively. Another study was conducted to increase the power generation in the BEF system by Zhuang *et al.* In this study, a two-chambered MFC separated by GORE-TEX cloth membrane, with a chamber fed by brewery effluent as a substrate, a CF anode electrode, and a cathode chamber with a Fe@Fe_2_O_3_/NCF composite electrode were used. The application of MFC system along with the Fenton's reaction resulted in a 4-fold increase in the power output capacity. The ˙OH production from cathode Fenton's reactions with high redox potential effectively increased the power generation.^86^

In order to achieve simultaneously the anodic oxidation of wastewater and the cathodic degradation of organic pollutants by the Fenton's reaction, Birjandi *et al.*^78^ conducted research and examined the feasibility of BEF system for medicinal herbs wastewater treatment and electricity generation. The BEF system was used with an anodic chamber equipped with graphite plate electrode, anaerobic sludge as an inoculum, medicinal herbs wastewater as substrate, and an aerobic cathode chamber filled with medicinal herbs wastewater and equipped with a Fe@Fe_2_O_3_/graphite composition as cathodic electrode. In a typical method of making the graphite/Fe@Fe_2_O_3_ composite electrode, different value of FeCl_3_·6H_2_O (0.7, 0.9 and 1 g) and also 1.8 g of NaBH_4_ were dissolved in 100 and 40 mL of distilled water, respectively. After the ultrasonic treatment of the graphite in the ferric solution for 20 min, the NaBH_4_ solution was slowly added to reduce ferric ions on the graphite. The composite electrode was washed with deionized water and dried for use in nitrogen gas. Under optimal conditions of BEF reactor with 0.9 g FeCl_3_–6H_2_O as iron source, the Nafion 112 as membrane and MLSS concentration of 3000 mg L^−1^, the maximum power density, coulombic efficiency, cathodic COD removal and anodic COD removal were 60.43 mW m^−2^, 4.09%, 84.02%, 78.05%, respectively. Moreover, the concentration of total iron ion and H_2_O_2_ at the steady level were 31 and 0.62 mg L^−1^, respectively within 70 h. The biological oxidation by anode microorganisms and the ˙OH production resulting from cathodic Fenton's reactions were the important reasons for the high degradation of environmental organic matter in the medicinal herbs wastewater. Xu *et al.*^100^ reported a similar case when using a new two-chambered BEF system with a FeVO_4_/CF combined cathode electrode for treating the coal gasification wastewater (CGW) without external power supply. In short circuit conditions, the residual concentrations of COD, TOC, BOD_5_, and total phenol in the cathode chamber effluent were determined to be 32.5 mg L^−1^, 8.8 mg L^−1^, 4.5 mg L^−1^, and 15.6 mg L^−1^, respectively that these concentrations were following the wastewater disposal standards. The advanced removal of a wide range of environmental persistent and toxic pollutants from CGW took place due to the production of strong oxidants such as ˙OH from Fenton-like reactions. In addition, the maximum power density and the current density were 849.7 mW m^−3^, 2.6 A m^−3^, respectively. In another study, the application of a two-chamber BEF-MFC system in the degradation of the oily wastewater at the cathode and dairy wastewater at the anode (as substrate and inoculum) using the FeSO_4_·7H_2_O and MnSO_4_·H_2_O catalysts was examined. The results showed that the BEF system using Fe^2+^ catalyst with the production of the maximum power density of 102 mW m^−2^, voltage of 0.3 V, and the COD removal rate of 40%/4 h had a better performance in comparison with the Mn^2+^ catalyst. Moreover, this system was introduced as an independent technology that did not need an external energy source.^176^

Generally, most BEF systems have used cation exchange membrane as separator, which has difficulty maintaining the low pH of the catholyte. Recent studies have identified the bipolar membrane as an effective separator that can prevent pH elevation in the cathode chamber and pH drop in anode chamber. Based on this point, Li *et al.* performed aniline (C_6_H_5_NH_2_) wastewater treatment using a MEC-Fenton system equipped with the bipolar membrane as an innovative BEF system and a promising alternative. This system was made with two anode and cathode chambers with the same volume of 100 mL and was equipped with a carbon fiber brush anode electrode and graphite plate cathode electrode. Aniline was degraded with removal efficiency of 97.1% under optimal conditions (pH 3, 10 mM Fe^2+^, 0.5 V applied voltage and 16 mL min^−1^ air flow rate) for 6 days. Moreover, the TOC removal efficiency was determined to 93.1% under these conditions.^177^

The landfill leachate with a complex nature contains a wide range of recalcitrant, very hazardous, stable, and toxic compounds that untreated landfill leachate using appropriate methods can enter to surface water, groundwater and soil.^99,178–180^ In an experiment conducted by a Li *et al.*^181^ the persistent compounds degradation of the landfill leachate was performed using the BEF system with a two-chamber MFC equipped with CF anode electrode and a graphite cathode electrode with natural pyrrhotite coating. The maximum power density of 4.2 W m^−3^ was produced using a pyrrhotite cathode electrode more than a graphite cathode electrode. In addition, the removal of 78% of COD and 77% of dye from the real landfill leachate after 45 days showed that pyrrhotite acted as a cost-effective Fenton catalyst in energy generation and advanced cathodic degradation of environmental organic contaminants in real leachate. Similarly, Hassan *et al.*^99^ was also reported the application of a two-chamber BEF system with CF anode and cathode electrodes to treat real landfill leachate effluent containing persistent organic compounds which were pre-treated using nitritation-anammox reactor. The performance of this system was evaluated using different sources of iron, including iron(iii) chloride hexahydrate and iron(ii) sulfate heptahydrate as Fenton catalysts. The iron(ii) sulfate concentration of 300 mg L^−1^ removed COD of 26.0 ± 9.3–33.6 ± 1.7% (BEF-1) and also 21.3 ± 3.2–40.7 ± 3.1% (BEF-2) in cycle 5–8. On the other hand, an average COD removal efficiency using iron(iii) chloride with same iron concentration was obtained 31.4 ± 12.2% (BEF-1) and 35.2 ± 13.8% (BEF-2) in cycle 9 which decreased 18–23% in cycle 10 and 11. In their study, the iron(ii) sulfate catalyst using Fenton's reactions showed slightly better COD removal efficiency in comparison with the iron(iii) chloride using Fenton like-reactions. This is because the ˙OH production in Fenton's reactions at the beginning of the reaction occurs faster than in Fenton-like reactions, and this may be due to the high rate constant of (eqn (4)) relative to (eqn (5)). In general, the anodic and cathodic COD removal efficiency were 71–76% and 77–81%, respectively, using glucose as anodic substrate and leachate as catholyte with 300 mg L^−1^ iron(ii) sulfate. After using the real leachate as anode substrate, the COD removal efficiency decreased. Nonetheless, the current density did not change significantly, and the maximum current density was determined 1.7 A m^−2^. In another study,^178^ this research group evaluated the performance of the new BEF system with a combination of anodic biooxidation and cathodic EF as post-treatment an anammox system. Their study focused on the landfill leachate treatment with environmental persistent organic pollutants containing 2401 ± 562 mg COD L^−1^ and 237 ± 57 mg BOD_5_ L^−1^. In this system, the removal rate of COD in the batch mode of BEF system was 318–351 mg L^−1^ d^−1^. Furthermore, in the continuous mode of this system, the removal rate of COD was 1077–1244 mg L^−1^ d^−1^ with simultaneous current density production of 43.5 ± 2.1 A m^−3^. Thus, this system combined with the anammox process, was introduced as an effective and suitable technology for advanced treatment of the landfill leachate.

The above discussed demonstrations prove that the BEF process is an effective and feasible alternative that has shown a successful performance by removing the environmental organic pollutants in the aquatic environments, treating wastewater, and generating bioenergy. Moreover, it can provide a potentially sustainable solution to the challenges of environmental pollution and can result in environmental remediation.

## 4. Challenges and future prospects associated with bio-electro-Fenton systems

The innovative technology of BEF is found to be a feasible and energy efficient solution for removing the environmental persistent pollutants and this issue has gained much attention of the researchers.^57,68,182^ The BEF as an emerging technology is bringing new opportunities. This technology has major advantages along with the integration of the MFC process's microbial metabolism to bioenergy production and the electrochemical reactions of the EF process to the wastewater treatment that contains environmental organic pollutants.^57,71,74^ The first advantage is minimizing the cost of supplying Fenton reagents (H_2_O_2_ and Fe^2+^) because of the *in situ* electrochemical production of H_2_O_2_ and Fe^2+^ which are needed for pollutants degradation. This advantage avoids the risk of transporting and storing chemicals and thus reduces the risk of accidents. Previous reports have shown that *in situ* generation of H_2_O_2_ can decrease the intense energy requirements. The second major advantage includes no external energy source requirement with low-cost sustainable bioenergy generation. This advantage is an appropriate solution to solve the high electricity consumption problem in the EF-based processes. Amenable to real-time monitoring with good operational stability is the third advantage of this process. Finally, the forth advantage is the use of this system as an environmentally friendly technology for treating wastewater.^35,48,56,94,103^

Nonetheless, practical application of BEF system has not been realized, because of some major challenges in cost and system development.^94^ To find out whether the expected benefits of this system can be eventually achieved, we need to be investigate and control the challenges and feasibility of this technology in order to implement and commercialize in environmental remediation. These important challenges need to be discussed.

One of the important operational problems in commercialization of BEF system is the decrease in the current density generation during scaling up of system. The isolation of the strong microorganisms, the production of recombinant engineered strains of bacteria, or the identification of new mediator-producing bacteria can effectively transfer electrons to the anode and increase the current density.^44,183^ In addition, electrodes are the habitat of exoelectrogenic microorganisms and affect the activity of microorganisms to improve the electron transfer capacity and the performance of the BEF process.^16,184^ Therefore, electrode materials must have a large surface area, high electrical conductivity, high stability, and strong surface biocompatibility. The modification of the anodic electrode using nanomaterial, such as carbon nanotube/polyaniline composite anode electrode, is an option to strengthen the electrode surface and electron transfer. In addition, the metallic nanoparticles have been reported to act as suitable linkers between the active site of the enzyme and the electrode, which can solve this problem. Moreover, the ionic liquid polymer coating on the carbon electrodes provides a new opportunity to produce high power densities and it has been reported that this coating improves bioenergy production by increasing the bacterial load capacities.^16,105,183,185,186^

The high cost of the some electrodes and membranes is one of the major problems that prevents from scaling up of this process.^187–189^ This particularly demands attention in developing the electrode materials and membrane. It should be sufficiently low. The use of low-cost, durable electrodes and membranes can affect the financial cost and long-term stability of the entire system. It seems that carbon materials are desirable choices due to their stability, biocompatibility, and high surface area. Moreover, CF, carbon mesh, carbon veil, granular activated carbon, and graphite plate and graphite rods are the types of inexpensive electrodes and high-quality commercially desirable products which can be used in BEFs. Notwithstanding, the comparison of different anode and cathode electrodes performance in regard to their effects on biofilm, electron transfer capacity, overall system performance and operating costs for commercialization should be examined in the future.^48,52,105,190,191^

Furthermore, membrane materials play an important role in the structure and function of BEF systems.^53,110^ However, the cation and proton exchange membranes are expensive, and the phenomenon of membrane pore fouling and blockage results from the long-term system activity.^48,192–194^ Therefore, the use of certain membranes such as clayware and ceramic membranes with high mechanical and chemical stability and easy availability^195,196^ and nano-composite membranes,^197^ non-woven fabric polypropyle membranes,^198^ and micro porous filtration membranes^188,199^ can also be alternatives to these expensive membranes and can be reduce the financial costs of the reactor. Nonetheless, the development of the various low-cost and anti-fouling membranes with the materials science advances is still necessary.^198,200,201^ Many researchers are trying to find the proper combination of high-performance, low-cost, and multi-purpose materials that can be used to improve system performance for commercialization and increase the economical scale in the future.^52,105^

The adjustment of the acidic pH of the cathode chamber is an important constraint on this process and results in additional operating costs.^35^ The use of heterogeneous Fenton catalysts, which increase the working range of pH, has been proposed as a choice.^202,203^ The other options for overcoming this limitation include the use of Fe-modified activated carbon electrodes,^204^ Fe-impregnated CF electrodes,^205^ graphene-based cathode electrodes,^206^ or CoFe-layered double hydroxide modified CF cathode electrodes,^207^ and other types of the modified cathode electrodes. However, these issues need further investigation.

In the BEFs, adding an iron-based catalyst and increasing the iron concentration result in the formation of some sludge.^73,90^ The use of homogeneous iron catalysts creates the problem of residual iron in the treated water and may increase the operating cost of the system.^130,146^

This problem can be solved by means of treatment and disposal operations.^170,208^ Residual sludge can be reduced by optimizing the amount of iron salt as a Fenton reagent and reusing the iron content from the sludge that careful attention is required to solve this problem. The use of some iron catalysts to achieve a catalytic regeneration of ferrous iron can prevent the iron sludge formation. Heterogeneous iron catalysts can be used to minimize the sludge generation in the BEF system.^132,146,209–211^ In the case of heterogeneous iron catalysts, natural iron oxide minerals, clays containing iron, zero-valent iron (Fe^0^), M-type strontium hexaferrite magnetic nanoparticles (SrM-NPs), and iron immobilized in solid supports have been utilized.^73,146^

During the last decades, Fenton like systems using non-iron based Fenton catalysts have also been developed. Cobalt, cerium, manganese, copper, and another redox metal oxides were utilized as catalysts to investigate their reactivity to H_2_O_2_ degradation into ˙OH in the Fenton-like degradation of organic pollutants.^212–215^ Cerium oxides (CeO_2_) (such as CeO_2_ nanorod and CeO_2_ nanocube) as heterogeneous Fenton-like catalysts have been attracted great interest for their catalytic oxidation applications.^216^ Additionally, Ce-doped MOF and CuO/Al_2_O_3_ are also efficient heterogeneous Fenton-like catalyst with high activity in Fenton-like oxidation of hazardous pollutants.^215,217^

Generally, BEF system can be a promising cost-effective technology for removing the environmental persistent pollutants and producing clean energy.^68^ It is expected that the use of large-scale industrial BEF processes will be an efficient system for treating wastewater that contains industrial pollutants. This issue stems from the fact that it can provide a certain amount of the electricity which is required by the wastewater treatment systems and undoubtedly has the potential to recover energy during wastewater treatment. Therefore, combining wastewater treatment with electricity generation can help to offset the costs of wastewater treatment and to make it more economical.^59,218^ In order to determine the practical implementation of this technology as a wastewater treatment system, these challenges must be resolved, and various studies must be conducted using real wastewaters. Furthermore, a number of strategies should be developed in order to improve the environmental persistent organic pollutants degradation and the energy production by controlling biological and electrochemical reactions.^53,171^ In addition, combining BEF technology with other conventional wastewater treatment technologies can increase the application and efficiency of this technology by creating a synergistic effect.^219^ Moreover, new designs and serious techno-economic analysis are needed in order to scale up of the system from a laboratory scale to full scale and to optimize and improve the system performance in real conditions. Most importantly, the capital costs of BEF system should be reduced to allow for the large-scale application of this system in order to bio-electrochemical wastewater treatment and the renewable power generation from wastewater. As a result, more research efforts are needed to promotion the performance of BEF and to develop a deeper understanding of BEF systems to enable process optimization.^59,110,171,220,221^

## 5. Conclusions

The present review examined the BEF system efficiency in regard to the degradation and mineralization of environmental organic pollutants by reviewing most of the existing studies. The study intended to inform the researchers about the existence of this bio-electrochemical process with desirable efficiency, which is effective in removing the environmental persistent compounds in wastewater and producing stable bioenergy. In this regard, the potential successful application of this technology regarding the removal of a wide range of environmental persistent organic pollutants such as different types of toxic and stable industrial dyes, pharmaceutical compounds, emerging pollutants, and the effective treatment of some real complex wastewaters has been proven. Furthermore, there have been significant technological advances in BEF processes in regard to the treatment of different environmental organic pollutants in wastewater from various sources and industries. This study examined the use of the BEF process for degrading environmental organic pollutants in the cathode and anode chambers, the bioenergy production, the analysis of the pollutants degradation efficiency, the impacts of important factors and the major advantages and disadvantages of the system. Considering the diversity of the environmental organic pollutants and the complexity of their structure, a number of these numerous challenges in regard to the improvement of the pollutant removal capacity, the mechanism of the aforementioned process, and the development of BEF systems have been considered in recent studies. Nonetheless, this issue requires more attention and should be considered in further research. On the other hand, the analysis of the results of recent studies shows that the proper selection of anodic and cathodic materials, pH, substrate source, inoculum, iron catalyst source, optimal production of H_2_O_2_, proper mode of operation, and improvement of BEF designs using cost-effective materials can be effective in obtaining a high power density value, a maximum removal efficiency of organic pollutants, and producing low-cost energy in this system. Consequently, the increase in the number of studies on the analysis of bio-electro-Fenton systems results in the rapid development of the design and improved the efficiency of this technology. Moreover, these studies provide a better perspective on developing an efficient and compatible platform for new and acceptable wastewater treatment technologies. We hope that the development of better studies, which examine the economic issues, the technology of bio-electrochemical systems, and the structure and mechanisms of the BEF system, will provide us with new opportunities in the future to develop this wastewater treatment technology. It is hoped that this technology will be a practical plan, among the other choices, for treating wastewater efficiently and producing sustainable bioenergy in the near future.

## Conflicts of interest

There are no conflicts to declare.

## Supplementary Material
